# Prescription opioid dispensing patterns among patients with schizophrenia or bipolar disorder

**DOI:** 10.1186/s12888-024-05676-5

**Published:** 2024-04-02

**Authors:** Brittany D. Roy, Jianheng Li, Cathy Lally, Sarah C. Akerman, Maria A. Sullivan, James Fratantonio, William Dana Flanders, Madé Wenten

**Affiliations:** 1 Alkermes, Inc., Waltham, MA USA; 2 Epidemiologic Research & Methods, LLC, Atlanta, GA USA

**Keywords:** Chronic pain, Mental disorders, Pain, Pharmacoepidemiology, Prescription drugs

## Abstract

**Background:**

Patients with schizophrenia (SZ) or bipolar disorder (BD) may have increased risk of complications from prescribed opioids, including opioid-induced respiratory depression. We compared prescription opioid pain medication dispensing for patients with SZ or BD versus controls over 5 years to assess dispensing trends.

**Methods:**

This retrospective, observational study analysed US claims data from the IBM® MarketScan® Commercial and Multi-State Medicaid databases for individuals aged 18–64 years with prevalent SZ or BD for years 2015–2019 compared with age- and sex-matched controls. Baseline characteristics, comorbidities, and medication use were assessed. Proportions of individuals dispensed prescription opioids chronically (ie, ≥70 days over a 90-day period or ≥ 6 prescriptions annually) or nonchronically (≥1 prescription, chronic definition not met) were assessed.

**Results:**

In 2019, the Commercial and Medicaid databases contained records for 4773 and 30,179 patients with SZ and 52,780 and 63,455 patients with BD, respectively. Patients with SZ or BD had a higher prevalence of comorbidities, including pain, versus controls in each analysis year. From 2015 to 2019, among commercially insured patients with SZ, chronic opioid-dispensing proportions decreased from 6.1% (controls: 2.7%) to 2.3% (controls: 1.2%) and, for patients with BD, from 11.4% (controls: 2.7%) to 6.4% (controls: 1.6%). Chronic opioid dispensing declined in Medicaid-covered patients with SZ from 15.0% (controls: 14.7%) to 6.7% (controls: 6.0%) and, for patients with BD, from 27.4% (controls: 12.0%) to 12.4% (controls: 4.7%). Among commercially insured patients with SZ, nonchronic opioid dispensing decreased from 15.5% (controls: 16.4%) to 10.7% (controls: 11.0%) and, for patients with BD, from 26.1% (controls: 17.5%) to 20.0% (controls: 12.2%). In Medicaid-covered patients with SZ, nonchronic opioid dispensing declined from 22.5% (controls: 24.4%) to 15.1% (controls: 12.7%) and, for patients with BD, from 32.3% (controls: 25.9%) to 24.6% (controls: 13.6%).

**Conclusions:**

The proportions of individuals dispensed chronic or nonchronic opioid medications each year were similar between commercially and Medicaid-insured patients with SZ versus controls and were higher for patients with BD versus controls. From 2015 to 2019, the proportions of individuals who were dispensed prescription opioids chronically or nonchronically decreased for patients with SZ or BD and controls.

**Supplementary Information:**

The online version contains supplementary material available at 10.1186/s12888-024-05676-5.

## Introduction

Opioid analgesic medications have a role in pain management; however, there are potentially serious harms associated with their use [1–3]. Also, there is limited evidence to support their benefit, compared with that of nonopioid medications for the long-term management of chronic noncancer pain [4]. Patients living with a serious mental illness (SMI) may have an increased risk of complications associated with opioid medications, including overdose and misuse [1, 5]. Significant associations with opioid overdose in patients with schizophrenia and in patients with bipolar disorder have been reported [6–8]. Despite this risk, prescription opioid dispensing and use have been reported to occur more frequently in patients diagnosed with SMI, particularly in individuals diagnosed with a mood disorder, compared with individuals without these disorders [9–12]. Patterns of prescription opioid use may differ by the underlying mental health diagnosis, although there is a relative lack of data examining opioid prescribing in patients with schizophrenia or bipolar disorder [12].

After years of increased opioid prescribing through the early 2000s, this trend slowed after 2010, likely due to reductions in prescribing high-dose opioids and a decline in the utilisation of opioids for long-term pain management [13–15], even as rates of opioid use disorder and opioid-related overdose deaths continued to rise [16–19]. Subsequently, increased restrictions and limitations placed on opioid prescribers, along with media attention regarding the opioid epidemic, likely contributed further to the reduction of prescription opioid dispensing overall. Efforts to further reduce risks associated with opioid prescribing included the 2016 publication of the US Centers for Disease Control and Prevention (CDC) guideline for prescribing opioids for chronic pain [1]; after its publication, opioid prescribing continued to decline for the general population [13, 20]. While trends over time in opioid prescribing have been examined more generally in the larger US population, trends among the populations at the highest risk for opioid-associated complications have not been as well studied [21], and opioid prescribing for individuals with schizophrenia or bipolar disorder has not been examined over extended time periods.

We conducted a retrospective, observational study to assess the temporal changes in patterns of prescription opioid dispensing over a 5-year period, beginning 1 year prior to publication of the CDC guideline (ie, 2015) through 2019. We assessed the prevalence of prescription opioid pain medication dispensing to individuals with schizophrenia or bipolar disorder compared with age- and sex-matched controls by using national health insurance claims data to determine any associations between these diagnoses and chronic or nonchronic opioid dispensing.

## Methods

### Study design and data sources

This retrospective study was conducted using de-identified, person-specific health insurance claims data from the IBM® MarketScan® Research Databases. The IBM® MarketScan® Commercial Database includes medical and prescription data from individuals in the United States covered by employer-sponsored private health insurance; the IBM® MarketScan® Multi-State Medicaid Database contains records of inpatient services and admissions, outpatient services, and prescription drug information for US Medicaid enrolees from multiple states.

**Fig. 1:**
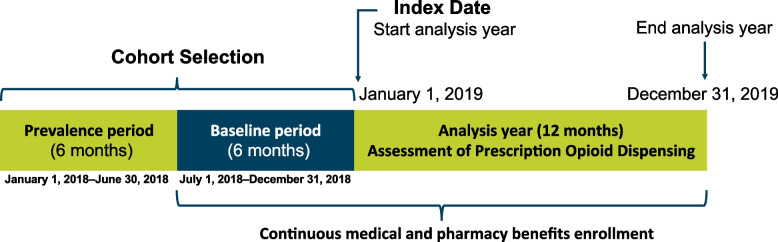
Study Design for Each Analytic Window (Illustrated Using the Example of Analysis Year 2019)

The study period spanned from January 1, 2014, to December 31, 2019, and included 5 analysis years (2015–2019). Each analysis year included an analytic window that was 24 months in duration, including 12 months prior to, and 12 months after, the index date (ie, “baseline” year and “analysis” year, respectively), defined as January 1 of the respective analysis year (Fig. 1). The baseline year immediately preceding the index date was utilized to select each cohort. Baseline demographic and clinical characteristics were assessed 6 months prior to the index date. The analysis year, starting on the index date, was used to identify annual prescription opioid dispensing (Fig. 1).

### Study population

Individuals aged 18 to 64 years on the index date of the analysis year, with at least 6 months’ continuous enrolment in medical and pharmacy benefits during the baseline period and continuous enrolment in medical and pharmacy benefits during the entire analysis year, were eligible for inclusion in the analysis for that year. Individuals who were dually eligible for Medicaid and Medicare (Medicaid database analyses only), who had a cancer or metastatic cancer diagnosis, or who had claims for palliative or hospice care during the 24-month analytic window, were excluded.

Patients were required to have at least 1 recorded inpatient or 2 outpatient medical claims on different dates for schizophrenia (International Classification of Diseases, 9th Revision, Clinical Modification [ICD-9-CM]: 295.xx; International Classification of Diseases, 10th Revision, Clinical Modification [ICD-10-CM]: F20.xx, F25.9) or bipolar disorder (ICD-9-CM: 296.xx, 301.13; ICD-10-CM: F31.x) during the baseline year. At least 1 claim was required to be recorded between January 1 and June 30 of the baseline year. If a patient had qualifying claims for both schizophrenia and bipolar disorder during the baseline year, they were included in the schizophrenia cohort only, using a hierarchical approach. This approach has been used in prior studies [12, 22], as there are diagnostic challenges with these disease states [23].

One control from the same database and analysis year was matched without replacement to each patient with schizophrenia or bipolar disorder based on their age at index and sex. Controls met the same enrolment criteria as patients with schizophrenia or bipolar disorder but had no claim for schizophrenia or bipolar disorder at any time during the 6-year period (January 1, 2014–December 31, 2019). Matched controls were selected for schizophrenia cases first, and then were removed from the pool prior to matching controls to the bipolar disorder cases.

### Variables

Demographic characteristics, comorbidities, and medication utilization were evaluated during the baseline period for each analysis year. Demographic characteristics included age and sex; additionally, the Commercial database included region, and the Medicaid database included race. Clinical comorbidities were assessed using the Charlson Comorbidity Index (CCI), a weighted score based on the number and seriousness of comorbid diseases [24]. Higher CCI scores are associated with a more severe burden of comorbidities and a greater 1-year risk of mortality. Comorbid pain diagnoses were identified by the presence of at least 2 ICD-9-CM/ICD-10-CM codes; psychiatric comorbidities were denoted by the presence of at least 1 ICD-9-CM/ICD-10-CM code. Medications of interest (antipsychotics, antidepressants, mood stabilizers, anticonvulsants, anxiolytics, and sedatives and hypnotics) with at least 1 prescription dispensed were recorded.

All prescription claims for opioids indicated for the treatment of pain were assessed for each analysis year. This analysis excluded opioid products provided for non–pain indications (eg, cough-cold medicine containing opioids, opioid-containing medications for the treatment of opioid use disorder). Formulations of methadone and buprenorphine dispensed for pain indications were included. Chronic prescription opioid dispensing was defined as coverage for at least 70 days in any 90-day period, or at least 6 prescriptions dispensed during the analysis year [12]. Nonchronic opioid dispensing was defined as at least 1 prescription dispensed in an analysis year but falling below the dispensing threshold that defined chronic.

### Statistical analysis

Demographic and clinical characteristics were summarized descriptively by group (schizophrenia, bipolar disorder, and matched controls), analysis year, and database (Commercial and Medicaid).

The proportion of individuals meeting the definition for chronic and nonchronic prescription opioid dispensing was calculated by year for each psychiatric diagnosis category and database, and was compared with controls. These proportions were plotted over time, and regression curves were fitted using joinpoint regression methodology. Trends over time were analysed using the Joinpoint Regression Program, version 4.9.0.0 (National Cancer Institute, USA) [25]. The statistical significance of a change of slope overall from 2015 to 2019 in either chronic or nonchronic prescription opioid dispensing and for any significant inflection points in the slope was assessed.

A post hoc analysis was conducted to assess the average annual total days of prescription opioid dispensing for patients with schizophrenia or bipolar disorder for each database and dispensing category (chronic or nonchronic) compared with that of the matched controls.

The odds of receiving chronic or nonchronic prescription opioid dispensing across the 5 analytic years was assessed for patients in each psychiatric disease category compared with matched controls using logistic regression models allowing for within-matched-pair correlation and adjusted for age, sex, race (in the Medicaid database only), analysis year, pain diagnoses, and medical comorbidities (CCI).

Sample selection and creation of analytic variables were performed using Instant Health Data (IHD) software (Panalgo, Boston, MA, USA). Statistical analyses were performed using R version 4.0.2 (R Foundation for Statistical Computing, Vienna, Austria) and SAS Enterprise Guide version 7.15 (SAS Institute Inc., Cary, NC, USA). Sensitivity analyses were conducted using less stringent eligibility criteria (≥1 month of enrolment beginning at the start of the analysis year) to determine whether continuous medical and pharmacy enrolment influenced the results of the main analyses.

## Results

### Study population

Sample sizes for the 5 analysis years are listed in Additional Files 1, 2, 3, 4 and 5. For the most recent analysis year (2019), the study sample included 4773 patients with schizophrenia and 52,780 patients with bipolar disorder in the Commercial database, and 30,179 and 63,455 patients, respectively, in the Medicaid database, with corresponding numbers of matched controls. Demographics and baseline characteristics for the 2019 analysis year are presented in Table 1, and for each of the 5 analysis years in Additional Files 1, 2, 3, 4 and 5. Among patients with schizophrenia, the median age at index date trended downward between 2015 and 2019 in both the Commercial and Medicaid databases. Other demographics remained generally stable over the 5-year period. No demographic trends over time were observed for patients with bipolar disorder. In the Medicaid database, race was unbalanced between individuals diagnosed with schizophrenia or bipolar disorder and controls (Table 1) and was, therefore, controlled for in the logistic regression analyses.

**Table 1:** Demographic and Clinical Characteristics: Patients With Schizophrenia or Bipolar Disorder and Matched Controls, 2019

**Commercial Database**^**a**^ **2019**				
**Parameter**	**Schizophrenia** **(*N*** **= 4773)**	**Schizophrenia Controls** **(*N*** **= 4773)**	**Bipolar Disorder** **(*N*** **= 52,780)**	**Bipolar Disorder Controls** **(*N*** **= 52,780)**
Age, mean (SD), years	39.0 (14.7)	39.0 (14.7)	41.5 (13.6)	41.5 (13.6)
Sex, *n* (%)				
Female	1998 (41.9)	1998 (41.9)	34,683 (65.7)	34,683 (65.7)
Male	2775 (58.1)	2775 (58.1)	18,097 (34.3)	18,097 (34.3)
Regional division (Commercial database only), *n* (%)				
East North Central	844 (17.7)	761 (15.9)	9544 (18.1)	8696 (16.5)
East South Central	254 (5.3)	245 (5.1)	3273 (6.2)	2863 (5.4)
Middle Atlantic	1047 (21.9)	672 (14.1)	8325 (15.8)	7762 (14.7)
Mountain	190 (4.0)	242 (5.1)	2724 (5.2)	2629 (5.0)
New England	165 (3.5)	153 (3.2)	2188 (4.2)	1780 (3.4)
Pacific	345 (7.2)	431 (9.0)	3571 (6.8)	4392 (8.3)
South Atlantic	890 (18.7)	942 (19.7)	10,002 (19.0)	10,575 (20.0)
West North Central	253 (5.3)	261 (5.5)	2721 (5.2)	2713 (5.1)
West South Central	331 (6.9)	551 (11.5)	4757 (9.0)	5668 (10.7)
Unknown	454 (9.5)	515 (10.8)	5675 (10.8)	5702 (10.8)
CCI^b,c^				
Mean (SD)	0.25 (0.70)	0.09 (0.41)	0.25 (0.69)	0.10 (0.43)
Non-CCI comorbidities, *n* (%)^c^				
Pain	1734 (36.3)	1215 (25.5)	26,064 (49.4)	16,038 (30.4)
Substance use disorder^d^	540 (11.3)	39 (0.8)	5570 (10.6)	357 (0.7)
Nicotine dependence	534 (11.2)	81 (1.7)	4406 (8.4)	949 (1.8)
Anxiety disorder	1409 (29.5)	289 (6.1)	21,942 (41.6)	4044 (7.7)
Depressive disorder	1146 (24.0)	224 (4.7)	14,641 (27.7)	3188 (6.0)
Post-traumatic stress disorder	269 (5.6)	15 (0.3)	4638 (8.8)	245 (0.5)
Attention-deficit/hyperactivity disorder	274 (5.7)	100 (2.1)	7760 (14.7)	944 (1.8)
Personality disorder	163 (3.4)	6 (0.1)	2103 (4.0)	35 (0.1)
**Medicaid Database**^**e**^ **2019**				
**Parameter**	**Schizophrenia** **(*N*** **= 30,179)**	**Schizophrenia Controls** **(*N*** **= 30,179)**	**Bipolar Disorder** **(*N*** **= 63,455)**	**Bipolar Disorder Controls** **(*N*** **= 63,455)**
Age, mean (SD), years	43.4 (12.8)	43.4 (12.8)	39.5 (12.4)	39.5 (12.4)
Sex, *n* (%)				
Female	12,135 (40.2)	12,135 (40.2)	44,925 (70.8)	44,925 (70.8)
Male	18,044 (59.8)	18,044 (59.8)	18,530 (29.2)	18,530 (29.2)
Race (Medicaid database only), *n* (%)				
Black	14,569 (48.3)	10,191 (33.8)	12,646 (19.9)	23,223 (36.6)
White	11,330 (37.5)	14,840 (49.2)	43,239 (68.1)	30,871 (48.7)
Hispanic	539 (1.8)	797 (2.6)	940 (1.5)	1927 (3.0)
Other	3295 (10.9)	3564 (11.8)	5875 (9.3)	6345 (10.0)
Unknown	446 (1.5)	787 (2.6)	755 (1.2)	1089 (1.7)
CCI^b,c^				
Mean (SD)	0.64 (1.21)	0.31 (0.86)	0.62 (1.09)	0.23 (0.71)
Non-CCI comorbidities, *n* (%)^c^				
Pain	14,486 (48.0)	9819 (32.5)	40,721 (64.2)	20,080 (31.6)
Substance use disorders^d^	7290 (24.2)	1959 (6.5)	17,049 (26.9)	3199 (5.0)
Nicotine dependence	9952 (33.0)	3820 (12.7)	22,134 (34.9)	6989 (11.0)
Anxiety disorders	8763 (29.0)	2896 (9.6)	29,037 (45.8)	6352 (10.0)
Depressive disorders	8223 (27.3)	2908 (9.6)	20,820 (32.8)	6297 (9.9)
Post-traumatic stress disorder	2997 (9.9)	458 (1.5)	12,986 (20.5)	1040 (1.6)
Attention-deficit/hyperactivity disorder	1338 (4.4)	391 (1.3)	8088 (12.8)	912 (1.4)
Personality disorders	1871 (6.2)	71 (0.2)	4557 (7.2)	157 (0.2)

^a^Race not available in the Commercial database

^b^CCI is a weighted score based on the number and the seriousness (scored 1–6) of comorbid diseases; higher scores are associated with a greater risk of mortality [24]

^c^Comorbidities were assessed by ≥1 ICD-9-CM or ICD-10-CM diagnosis code for the particular condition occurring during the baseline period, except for pain (≥2 diagnosis codes)

^d^Not including nicotine dependence

^e^Regional division not available in the Medicaid database

*CCI* Charlson Comorbidity Index

In each database, patients with schizophrenia or bipolar disorder were more likely to have medical and psychiatric comorbidities, including pain, compared with controls (Table 1; Additional Files 1, 2, 3, 4 and 5). Although mean CCI scores were low in all cohorts because of the exclusion of patients with cancer diagnoses from the analysis along with the short evaluation period (6 months), they were numerically higher for patients diagnosed with schizophrenia or bipolar disorder compared with matched controls in each year and in both databases.

Pain was the most common comorbidity across all groups in both databases. In the Commercial database in 2019, the proportion of individuals with schizophrenia with pain diagnoses was 36.3% (controls: 25.5%) and 48.0% in the Medicaid database (controls: 32.5%). In 2019, the proportion of individuals with bipolar disorder and pain diagnoses was 49.4% in the Commercial database (controls: 30.4%) and 64.2% in the Medicaid database (controls: 31.6%). Across all analysis years, the proportions were highest in individuals with bipolar disorder and for those in the Medicaid versus Commercial database. Anxiety disorders and depressive disorders were also common among patients with schizophrenia or bipolar disorder in both databases (Table 1). Clinical characteristics were consistent between 2015 and 2019 (Additional Files 1, 2, 3, 4 and 5).

### Opioid dispensing patterns

#### Chronic prescription opioid dispensing

**Fig. 2:**
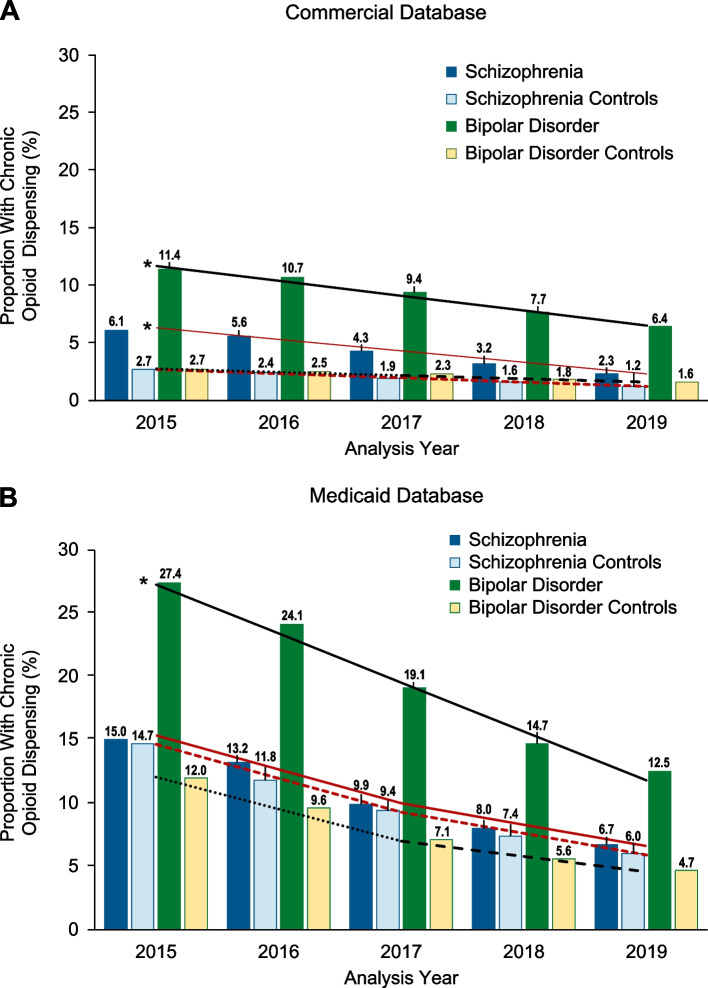
Proportion With Chronic Dispensing. A statistically significant decreasing trend (*P* < 0.05) in the proportion of individuals dispensed opioids chronically from 2015 to 2019 was observed in patients with schizophrenia, in patients with bipolar disorder, and in associated matched controls. **A** In the Commercial database, the slope of the joinpoint regression line was − 1.01 in patients with schizophrenia (solid red line) and − 0.37 in matched controls (dashed red line); the slopes differed significantly (ie, the hypothesis that the regression lines were parallel was rejected; *P* = 0.03). The slopes of the joinpoint regression lines in patients with bipolar disorder (− 1.30; solid black line) and in their matched controls (− 0.29; dashed black line) were also significantly different (*P* = 0.0002). **B** In the Medicaid database, a joinpoint was identified in 2017 in the regression lines for patients with schizophrenia and for controls; the slopes for the 2 cohorts did not differ (2015 to 2017: slope = − 2.68 and 2017 to 2019: slope = − 1.69; *P* = 0.78). For patients with bipolar disorder, the slope was −3.86 from 2015 to 2019, which differed significantly from their matched controls (2015 to 2017: slope = −2.53 and 2017 to 2019: −1.21, with a joinpoint at 2017; *P* = 0.03). *Slope significantly different from control

Joinpoint regression analysis revealed decreasing trends in chronic prescription opioid dispensing between 2015 and 2019 for patients with schizophrenia and their controls in both the Commercial and Medicaid databases (all *P* < 0.05; Fig. 2). In the Commercial database, the proportion of individuals diagnosed with schizophrenia who received chronic prescription opioid dispensing in 2015 was 6.1% and decreased over the 5 analysis years by 62%, to 2.3% in 2019. The proportion of patients with schizophrenia who had chronic prescription opioid dispensing was approximately twice that of their matched controls (controls: 2.7% in 2015, 1.2% in 2019). However, the decrease in chronic prescription opioid dispensing from 2015 to 2019 was significantly greater for patients with schizophrenia compared with controls (schizophrenia: slope = − 1.01; controls: slope = − 0.37; *P* = 0.03; Fig. 2A**)**.

Overall, a greater proportion of individuals received chronic prescription opioid dispensing in the Medicaid database compared with the Commercial database (Fig. 2). The proportion of Medicaid patients with schizophrenia who received chronic prescription opioid dispensing was 15.0% in 2015 and decreased by 55% to 6.7% in 2019. The proportion of control individuals on Medicaid who had chronic prescription opioid dispensing was similar (controls: 14.7% in 2015, 6.0% in 2019) and decreased with the same slope for patients with schizophrenia and controls; the decline in both groups was more rapid from 2015 to 2017 (schizophrenia and controls: slope = − 2.68) and slower from 2017 to 2019 (slope = − 1.69; *P* = 0.78; Fig. 2B).

Chronic prescription opioid dispensing also declined from 2015 to 2019 in patients diagnosed with bipolar disorder and their matched controls (all *P* < 0.05; Fig. 2). In the Commercial database, the proportion of individuals with bipolar disorder who had chronic prescription opioid dispensing decreased by 43%, from 11.4% in 2015 to 6.4% in 2019. Chronic prescription opioid dispensing was approximately 4 times higher in patients with bipolar disorder compared with controls in this database (controls: 2.7% in 2015; 1.6% in 2019). Again, however, the decrease in chronic prescription opioid dispensing over the 5 analysis years was significantly more rapid for patients diagnosed with bipolar disorder compared with their matched controls (bipolar disorder: slope = − 1.30; controls: slope = − 0.29; *P* = 0.0002; Fig. 2A).

The proportion of individuals with bipolar disorder who were dispensed chronic prescription opioids was also greater in the Medicaid database compared with the Commercial database but decreased across each of the 5 analysis years. Chronic prescription opioid dispensing in Medicaid-covered patients with bipolar disorder decreased by 54%, from 27.4% in 2015 to 12.4% in 2019. The proportion of patients with bipolar disorder who were dispensed prescription opioids chronically was more than 2 times greater among individuals diagnosed with bipolar disorder compared with controls (controls: 12.0% in 2015, 4.7% in 2019). From 2015 to 2019, the decrease in chronic prescription opioid dispensing over time was significantly greater for Medicaid patients with bipolar disorder (bipolar disorder: slope = − 3.86) versus their controls (*P* = 0.03); controls had a greater decrease between 2015 and 2017 (slope = − 2.53) than between 2017 and 2019 (slope = − 1.21) (Fig. 2B).

#### Nonchronic prescription opioid dispensing

**Fig. 3:**
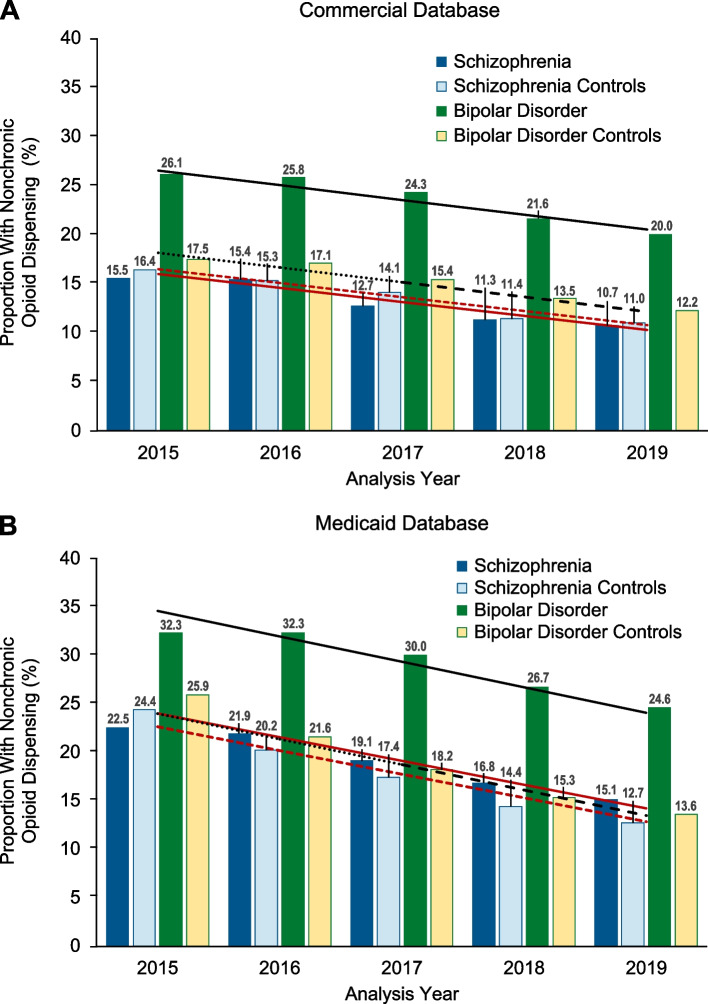
Proportion With Nonchronic Dispensing. A statistically significant decreasing trend (*P* < 0.05) in the proportion of individuals dispensed opioids nonchronically from 2015 to 2019 was observed in patients with schizophrenia, in patients with bipolar disorder, and in associated matched controls. **A** In the Commercial database, the slope of the joinpoint regression line was − 1.42 for both patients with schizophrenia (solid red line) and their matched controls (dashed red line; *P* = 0.43); the slope of the joinpoint regression line was − 1.50 for both patients with bipolar disorder (solid black line) and their matched controls (dashed black line; *P* = 0.10). **B** In the Medicaid database, the slope of the joinpoint regression line was − 2.45 for patients with schizophrenia (solid red line) and their controls (dashed red line; *P* = 0.23); among patients with bipolar disorder (solid black line) and their controls, the slope was − 2.63 (dashed black line; *P* = 0.10)

Nonchronic prescription opioid dispensing also decreased significantly from 2015 to 2019 for each analysis population across databases (all *P* < 0.05; Fig. 3). For patients with schizophrenia in the Commercial database, nonchronic prescription opioid dispensing decreased by 31%, from 15.5% in 2015 to 10.7% in 2019. The proportions of individuals with nonchronic prescription opioid dispensing were similar between patients with schizophrenia and controls (controls: 16.4% in 2015, 11.0% in 2019), and the decrease in dispensing over time did not differ between those cohorts (schizophrenia and controls: slope = − 1.42; *P* = 0.43; Fig. 3A).

Across cohorts, a higher proportion of patients in the Medicaid database were dispensed opioids chronically compared with those in the Commercial database (Fig. 3). In the Medicaid database, nonchronic prescription opioid dispensing decreased by 33% in patients with schizophrenia, from 22.5% in 2015 to 15.1% in 2019. The proportions of patients with nonchronic prescription opioid dispensing was comparable in schizophrenia controls from the Medicaid database (controls: 24.4% in 2015, 12.7% in 2019) and decreased similarly over the 5 analysis years (schizophrenia and controls: slope = − 2.45; *P* = 0.23; Fig. 3B).

For patients with bipolar disorder in the Commercial database, nonchronic prescription opioid dispensing decreased by 23%, from 26.1% in 2015 to 20.0% in 2019, and remained higher in patients with bipolar disorder versus controls over the 5 analysis years (controls: 17.5% in 2015, 12.2% in 2019). The decrease in prescription opioid dispensing over time did not significantly differ between the 2 cohorts (bipolar disorder and controls: slope = − 1.50; *P* = 0.10; Fig. 3A).

In the Medicaid database, nonchronic prescription opioid dispensing decreased by 24%, from 32.3% in 2015 to 24.6% in 2019 for patients with bipolar disorder. The proportion of patients dispensed prescription opioids nonchronically was approximately 6 to 12 percentage points higher for patients with bipolar disorder compared with controls in each analysis year (controls: 25.9% in 2015, 13.6% in 2019); however, the decrease in nonchronic prescription opioid dispensing over time was similar across patient and control groups (bipolar disorder and controls: slope = − 2.63; *P* = 0.13; Fig. 3B).

### Average annual days dispensed

The total average annual number of days for which individuals were prescribed opioids chronically generally increased over time in all cohorts and in both databases (Additional File 6). For patients with schizophrenia, the total average annual number of days of chronic prescription opioid dispensing increased from 237 days in 2015 to 247 days in 2019 in the Commercial database. In the Medicaid database, the increase was smaller, from 210 days in 2015 to 215 days in 2019. The average total number of days with chronic opioid dispensing was similar between individuals with schizophrenia and their associated controls across the 5 analysis years in the Commercial database (controls: 2015, 231 days; 2019, 252 days), but was lower for individuals with schizophrenia compared with their matched controls for each year in the Medicaid database (controls: 2015, 242 days; 2019, 254 days).

The total average annual number of days for which individuals with bipolar disorder were dispensed prescription opioids chronically increased over time in both the Commercial (from 228 days in 2015 to 242 days in 2019) and Medicaid databases (from 216 days in 2015 to 231 days in 2019). The average total number of days opioids were dispensed for individuals with bipolar disorder was similar to that of the matched controls across the analysis years in the Commercial database (controls: 2015, 224 days; 2019, 242 days) but was lower than that of controls across the analysis years in the Medicaid database (controls: 2015, 234 days; 2019, 249 days).

**Fig. 4:**
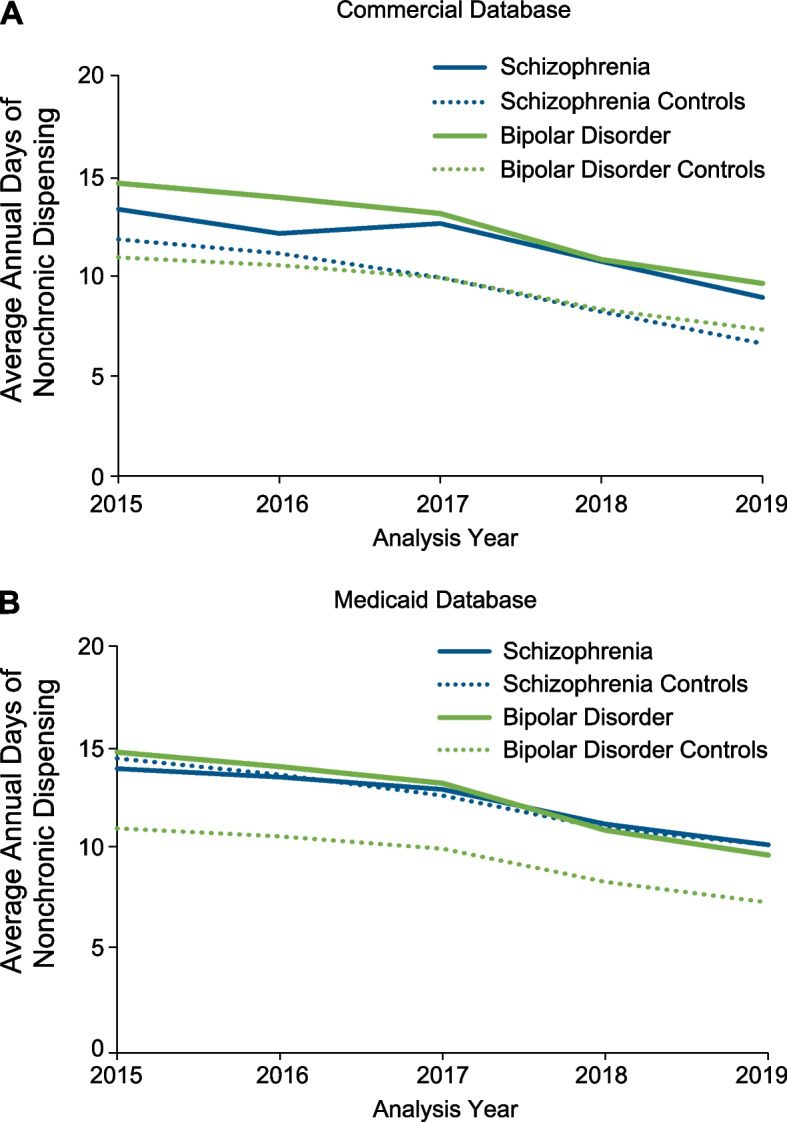
Average Annual Days of Dispensed Nonchronic Prescription Opioid Medications: **A** Commercial and **B** Medicaid Databases. Average annual number of days opioids were dispensed nonchronically declined in both the Commercial and Medicaid databases from 2015 to 2019 among patients with schizophrenia and those with bipolar disorder and their respective controls

The average annual number of days that nonchronic prescription opioids were dispensed decreased between 2015 to 2019 in all groups across both databases (Fig. 4). Individuals diagnosed with schizophrenia and bipolar disorder were dispensed nonchronic opioids for fewer than 15 days on average in each analysis year, with similar numbers of days dispensed for controls. By 2019, the average annual number of days dispensed was 10 days or fewer across all groups.

### Association between prescription opioid dispensing and schizophrenia or bipolar disorder

While chronic prescription opioid dispensing was relatively low in all groups in the Commercial database (Fig. 2), individuals with schizophrenia were 80% more likely to be dispensed chronic prescription opioids compared with matched controls after adjusting for relevant confounders, including pain diagnoses (odds ratio [OR]: 1.8; *P* < 0.05; Table 2). By contrast, the likelihood of chronic prescription opioid dispensing in the Medicaid database were 20% lower for patients with schizophrenia versus controls (OR: 0.8; *P* < 0.05; Table 2). After adjusting for confounding factors, patients with bipolar disorder in the Commercial database were 270% more likely than controls (OR: 3.7; *P* < 0.05), and those with bipolar disorder in the Medicaid database were 100% more likely than controls (OR: 2.0; *P* < 0.05), to have chronic prescription opioids dispensed (Table 2).

**Table 2:** Odds Ratios for Prescription Opioid Dispensing: Patients With Schizophrenia or Bipolar Disorder Versus Matched Controls

Parameter	Chronic Dispensing Adjusted Odds Ratio (95% CI)	Nonchronic Dispensing Adjusted Odds Ratio (95% CI)
Commercial database^a^		
Schizophrenia	1.8 (1.7, 2.0)^b^	0.9 (0.9, 0.9)^b^
Bipolar disorder	3.7 (3.6, 3.8)^b^	1.7 (1.7, 1.7)^b^
Medicaid database^a^		
Schizophrenia	0.8 (0.8, 0.8)^b^	0.9 (0.9, 0.9)^b^
Bipolar disorder	2.0 (1.9. 2.0)^b^	1.7 (1.6, 1.7)^b^

Analysis was adjusted for relevant confounders

^a^The odds of receiving chronic or nonchronic prescription opioid dispensing were assessed for each psychiatric disease compared with matched controls using conditional logistic regression models. Models assessing the Commercial database were adjusted for age, sex, year, pain diagnoses, and medical comorbidities (based on CCI); models assessing the Medicaid database were adjusted for the same factors, with the addition of race

^b^*P* < 0.05

*CCI* Charlson Comorbidity Index, *CI* confidence interval

The adjusted odds of nonchronic prescription opioid dispensing were 10% lower for patients with schizophrenia compared with that for controls in both the Commercial and Medicaid databases (both, OR: 0.9; *P* < 0.05). For patients with bipolar disorder, nonchronic prescription opioid dispensing was 70% more likely compared with controls (both databases, OR: 1.7; *P* < 0.05).

The results of sensitivity analyses using less stringent eligibility criteria supported, and were consistent with, the main findings.

## Discussion

To our knowledge, this analysis is the first to examine patterns of opioid pain medication dispensing for patients with schizophrenia or bipolar disorder over time. The 5-year period of analysis enabled assessment of opioid dispensing both before and after the 2016 publication of the CDC guideline for prescribing opioids for chronic pain [1]. The proportion of individuals dispensed chronic or nonchronic prescription opioids decreased significantly between 2015 and 2019 for patients with schizophrenia or bipolar disorder and their associated matched controls in the IBM® MarketScan® Commercial and Multi-State Medicaid Databases. By 2019, the proportion of individuals with schizophrenia dispensed opioids chronically was < 7% across databases with nonchronic opioid dispensing estimates of ≤15% across databases. Results were generally similar for matched controls. The proportions of individuals with bipolar disorder who were dispensed chronic or nonchronic prescription opioids were higher compared with their associated controls in each year. Chronic opioid dispensing proportions were greater in individuals with bipolar disorder compared with those with schizophrenia, indicating that there may be differences in prescribing across diagnostic categories of SMI, which is consistent with findings from earlier studies conducted with data from 2016 in the United States and from 2007 to 2014 in Sweden [12, 26]. Despite a higher proportion of dispensing for those with bipolar disorder, the average total annual days that prescription opioids were dispensed nonchronically did not differ substantially from those with schizophrenia, indicating exposure for similar lengths of time. The average annual number of days of nonchronic prescription opioid dispensing was relatively low in 2019, at no more than 10 days annually for both the schizophrenia and bipolar disorder groups.

Multiple federal and state initiatives targeting opioid prescribing practices [27, 28] have likely contributed to the general decline in opioid prescribing [29–32], which began prior to the first year of this analysis and continued throughout the remainder of the most recent analysis year [13–15, 33–35]. The high prevalence of prescription opioid dispensing noted in patients with bipolar disorder may be attributed in part to the sedative and antipsychotic properties of opioids [36]. The downward trend in prescription opioid dispensing in patients with schizophrenia or bipolar disorder observed in this study is consistent with declining opioid prescribing observed in the general population [13–15, 33–35]. The more rapid decline in chronic prescription opioid dispensing for most patient groups versus controls suggests that clinicians may have become more cautious in terms of opioid dispensing to patients with SMI. Although opioid prescribing declined more rapidly in the general population after the 2016 publication of the CDC guideline [13, 37], the current joinpoint analysis indicated that the decline in opioid dispensing was relatively consistent over time, suggesting that the guideline did not spur further changes among clinicians who were already reducing opioid dispensing to these patients. Given that the updated CDC guideline on prescribing opioids has been published [20], future research may reveal different patterns of opioid prescribing.

Some studies suggest that individuals with schizophrenia may have a reduced sensitivity to, or a disturbed or distorted experience of, pain compared with people without schizophrenia and, therefore, may not consistently report pain to their health care providers [38–43]. A previously published electronic medical record database study reported that fewer patients with schizophrenia had comorbid chronic pain diagnoses compared with controls in adjusted models, and the odds of chronic prescription opioid dispensing were similar between groups after adjusting for pain diagnoses and other relevant confounders [12]. In the current analysis, however, patients with schizophrenia were more likely than controls to have noncancer pain diagnoses but were significantly less likely to receive chronic opioid dispensing in the Medicaid database or nonchronic dispensing in either database. A schizophrenia diagnosis was associated with chronic prescription opioid dispensing in the Commercial database only. This pattern may be due to prescribing practices: clinicians may be less likely to identify a need for prescription pain medication in these patients [44].

The higher proportion of individuals with bipolar disorder receiving chronic and nonchronic prescription opioid dispensing compared with controls in the Commercial database analysis is notable but consistent with increased opioid prescribing observed for patients with versus those without mood disorders in published literature [9, 12, 45–47]. High rates of pain diagnoses have been reported among patients with bipolar disorder [12, 43, 48], and indeed in the current analysis, the proportions of individuals with pain diagnoses were higher for both cohorts, but particularly for those with bipolar disorder (49–68%; schizophrenia: 35–50%), compared with their respective matched controls (25–44%). These reported rates of comorbid pain fall within the wide prevalence ranges reported in meta-analyses (schizophrenia: 2–80% [pooled prevalence: 35%] [47]; bipolar disorder: 2–70% [pooled prevalence: 29%] [46]) and are consistent with rates of “any pain” reported by Owen-Smith et al. [12] (schizophrenia: 47%; bipolar disorder: 62%; matched controls: 42 and 40%, respectively). The significant association between bipolar disorder and both chronic and nonchronic opioid dispensing remained apparent after adjusting for the proportion of patients who had pain diagnoses. Although the reasons for the proportionally higher opioid dispensing in patients with bipolar disorder are unknown, it may be related to heightened pain sensitivity or disability associated with pain in patients with mood disorders [49, 50] or to the complex relationship between opioids and mood symptoms. Opioid use has been associated with antidepressant and anxiolytic effects in some patients [51–55] and with the exacerbation of mood symptoms in others [56–58].

Additional analysis of patient subgroups might provide insight into factors associated with differences in opioid dispensing among individuals with schizophrenia or bipolar disorder. For example, some nonopioid pain medications (eg, tricyclic antidepressants, serotonin-norepinephrine reuptake inhibitors, gabapentin, pregabalin) are also efficacious for treating neuropathic pain [59–61] and are recommended as first-line analgesics in its management [62, 63]. Approximately 46% to 64% of individuals with schizophrenia or bipolar disorder in the current analysis were prescribed an antidepressant at baseline. Although this was not part of the analysis, one might expect that those individuals—or the subset of those who received nonopioid pain medications for pain—have lower rates of opioid dispensing, on average, compared with those not taking these medications. Future research should also aim to further understand the effect of prescribing opioid analgesic medications on patient-centred outcomes in individuals with schizophrenia or bipolar disorder.

The 2016 CDC guideline for prescribing opioids for chronic pain and the updated 2022 guideline acknowledge that patients with SMI may be at higher risk of complications from opioid use and provide guidance for mitigating these risks, including approaches such as frequent re-evaluation of patients prescribed opioids and optimization of mental health treatment through the collaboration of pain and mental health treatment providers [1, 20]. Patients with SMI also may be more likely to be prescribed medications that could interact with opioid treatment (eg, benzodiazepines) [1, 20]. The guideline recommends that clinicians review state prescription drug monitoring program data to determine whether a patient is receiving opioid dosages or combinations that put the patient at high risk for overdose; safety concerns resulting from this review should be discussed with the patient. Additionally, utilizing toxicology tests are recommended in cases of suspected diversion to help ascertain if prescription opioids can be discontinued without precipitating opioid withdrawal. Lastly, naloxone should be offered to those patients at high risk [20]. Thus, the guideline recommends that clinicians carefully weigh the anticipated benefits of prescribing opioids against the risks to the patient and take steps that mitigate those risks [20].

### Limitations

Several limitations of the analysis should be noted. Patients were categorized diagnostically using a hierarchical approach. If a patient had qualifying claims for both schizophrenia and bipolar disorder, then the patient was included in the schizophrenia cohort only. This approach may have affected the data derived from the bipolar disorder cohort. This analysis assessed medications dispensed and could not address actual usage. Also, the results may underestimate total medications dispensed, as inpatient prescriptions are not reported in the databases utilized. Patients included in the IBM® MarketScan® Commercial and Multi-State Medicaid Databases may not be representative of patients with schizophrenia or bipolar disorder in the full US population, and findings may not be generalizable to those populations on Medicare, those who are dually eligible for Medicaid and Medicare, or to those individuals who are uninsured. The analysis was based on claims data from patients who met continuous medical and pharmacy enrolment criteria (6 months prior to January 1 of the analysis year and 12 months during the analysis year), although the sensitivity analysis results indicated that the study findings are not specific to individuals meeting the enrolment criteria used in the planned study design. Further, baseline clinical characteristics and diagnoses for this analysis were based on the ICD codes used in insurance claims for reimbursement, and not on formal clinician assessment. The sample size of most cohorts decreased from 2015 to 2019, likely as a function of the availability of commercial healthcare plans and various states’ Medicaid data availability in the IBM® MarketScan® research databases. Finally, the focus of this analysis was on prescription opioid medications indicated for the treatment of pain, given their potential for abuse or misuse. This approach captured most prescription opioid analgesic medications while excluding opioid products with lower utilisation (eg, cough-cold medicine containing opioids, medications containing opioids for the treatment of opioid use disorder). In this analysis, comorbid substance use disorder as a larger category was reported in 9% to 27% of individuals with schizophrenia or bipolar disorder, and therefore some portion of those whose data were included in this analysis may have received opioid-containing medication for that indication. However, this information cannot be fully captured in prescription claims data, as methadone dispensing through methadone maintenance treatment programs is not included in these data sources. Patients additionally may have been exposed to illicit opioids; however, that consideration is outside the scope of this analysis.

## Conclusions

The proportions of individuals dispensed prescription opioid pain medications, either chronically or nonchronically, decreased significantly from 2015 to 2019 for patients with schizophrenia, patients with bipolar disorder, and their matched controls in this analysis. Reasons for the reduction in prescription opioid dispensing over time likely include federal and state initiatives that have been put into place, including limitations on opioids dispensed, as well as heightened awareness of the opioid epidemic and its consequences. Despite the downward trend in opioid dispensing over time in each cohort examined, the proportions of both chronic and nonchronic opioid dispensing were higher for patients with bipolar disorder compared with matched controls and patients with schizophrenia. While the overall pattern of prescription opioid dispensing is declining, treating clinicians should consider that patients with schizophrenia or bipolar disorder may be at higher risk for complications from opioid use or may be prescribed medications concurrently that interact with opioids, emphasizing the importance of prescriber awareness and patient education [1, 5].

### Supplementary Information

**Additional File 1.** Demographic and Clinical Characteristics for Patients With Schizophrenia or Bipolar Disorder and Matched Controls: 2015.

**Additional File 2.** Demographic and Clinical Characteristics for Patients With Schizophrenia or Bipolar Disorder and Matched Controls: 2016.

**Additional File 3.** Demographic and Clinical Characteristics for Patients With Schizophrenia or Bipolar Disorder and Matched Controls: 2017.

**Additional File 4.** Demographic and Clinical Characteristics for Patients With Schizophrenia or Bipolar Disorder and Matched Controls: 2018.

**Additional File 5.** Demographic and Clinical Characteristics for Patients With Schizophrenia or Bipolar Disorder and Matched Controls: 2019.

**Additional File 6.** Average Annual Days of Chronic Prescription Opioid Dispensing: (A) Commercial Database. (B) Medicaid Database.

## Data Availability

All data generated or analysed during this study are included in this published article and its supplementary information files.

## Abbreviations

CCI: Charlson Comorbidity Index
CDC: Centers for Disease Control and Prevention
ICD-9-CM: International Classification of Diseases, 9th Revision, Clinical Modification
ICD-10-CM: International Classification of Diseases, 10th Revision, Clinical Modification
OR: Odds ratio
SMI: Serious mental illness
