# Supplementary material for: Prescription opioid dispensing patterns among patients with schizophrenia or bipolar disorder
Source: BMC Psychiatry. 2024 Apr 2;24:244. doi: 10.1186/s12888-024-05676-5 (PMC10986122; doi:10.1186/s12888-024-05676-5)
Supplement: Supplementary file 1 — Additional File 1. Demographic and Clinical Characteristics for Patients With Schizophrenia or Bipolar Disorder and Matched Controls: 2015. [file 12888_2024_5676_MOESM1_ESM.pdf]

Additional File 1. Demographic and Clinical Characteristics for Patients With Schizophrenia or Bipolar Disorder and Matched Controls: 2015

	Commercial Database[a] 2015				Medicaid Database[b] 2015			
Parameter	Patients With Schizophrenia (N=7784)	Schizophrenia Controls (N=7784)	Patients With Bipolar Disorder (N=58,200)	Bipolar Disorder Controls (N=58,200)	Patients With Schizophrenia (N=36,286)	Schizophrenia Controls (N=36,286)	Patients With Bipolar Disorder (N=49,734)	Bipolar Disorder Controls (N=49,734)
Age, mean (SD)	41.8 (14.6)	41.8 (14.6)	42.7 (13.6)	42.7 (13.63)	44.1 (12.5)	44.1 (12.5)	39.0 (12.4)	39.0 (12.4)
Median (Q1–Q3)	44 (26–55)	44 (26–55)	45 (32–54)	45 (32–54)	46 (33–55)	46 (33–55)	38 (29–49)	38 (29–49)
Age category, n (%)								
18–30 years	2441 (31.4)	2441 (31.4)	13,773 (23.7)	13,773 (23.7)	6842 (18.9)	6842 (18.9)	14,300 (28.8)	14,300 (28.8)
31–45 years	1702 (21.9)	1702 (21.9)	16,813 (28.9)	16,813 (28.9)	11,014 (30.4)	11,014 (30.4)	18,772 (37.7)	18,772 (37.7)
46–60 years	2866 (36.8)	2866 (36.8)	22,511 (38.7)	22,511 (38.7)	15,579 (42.9)	15,579 (42.9)	14,990 (30.1)	14,990 (30.1)
61–64 years	775 (10.0)	775 (10.0)	5103 (8.8)	5103 (8.8)	2851 (7.9)	2851 (7.9)	1672 (3.4)	1672 (3.4)
Sex, n (%)								
Female	3623 (46.5)	3623 (46.6)	37,881 (65.1)	37,881 (65.1)	16,637 (45.9)	16,637 (45.9)	35,309 (71.0)	35,309 (71.0)
Male	4161 (53.5)	4161 (53.5)	20,319 (34.9)	20,319 (34.9)	19,649 (54.2)	19,649 (54.2)	14,425 (29.0)	14,425 (29.0)
Race (Medicaid database only), n (%)								
Black	—	—	—	—	16,929 (46.7)	10,412 (28.7)	8,986 (18.1)	16,091 (32.4)

White	—	—	—	—	14,416 (39.7)	17,782 (49.0)	34,591 (69.6)	24,219 (48.7)
Hispanic	—	—	—	—	571 (1.6)	788 (2.2)	697 (1.4)	1393 (2.8)
Other	—	—	—	—	4370 (12.0)	7304 (20.1)	5460 (11.0)	8031 (16.2)
Regional division (Commercial database only), n (%)								
East North Central	1461 (18.8)	1170 (15.0)	9420 (16.2)	8927 (15.3)	—	—	—	—
East South Central	478 (6.1)	604 (7.8)	4682 (8.0)	4864 (8.4)	—	—	—	—
Middle Atlantic	1795 (23.1)	1160 (14.9)	9936 (17.1)	8554 (14.7)	—	—	—	—
Mountain	390 (5.0)	548 (7.0)	4030 (6.9)	3979 (6.8)	—	—	—	—
New England	322 (4.1)	309 (4.0)	2420 (4.2)	2074 (3.6)	—	—	—	—
Pacific	747 (9.6)	828 (10.6)	6196 (10.7)	6149 (10.6)	—	—	—	—
South Atlantic	1513 (19.4)	1809 (23.2)	12,523 (21.5)	13,223 (22.7)	—	—	—	—
West North Central	353 (4.5)	331 (4.3)	2639 (4.5)	2810 (4.8)	—	—	—	—
West South Central	690 (8.9)	985 (12.7)	6111 (10.5)	7304 (12.6)	—	—	—	—
Unknown	35 (0.5)	40 (0.5)	243 (0.4)	316 (0.5)	—	—	—	—

CCI[c,d]					—	—	—	—
Mean (SD)	0.27 (0.7)	0.09 (0.4)	0.24 (0.7)	0.10 (0.4)	0.58 (1.1)	0.37 (0.9)	0.59 (1.0)	0.28 (0.8)
Median (Q1–Q3)	0 (0–0)	0 (0–0)	0 (0–0)	0 (0–0)	0 (0–1)	0 (0–0)	0 (0–1)	0 (0–0)
Individual comorbidities included in the Charlson Comorbidity Index, n (%)								
AIDS/HIV	28 (0.4)	4 (0.1)	180 (0.3)	68 (0.1)	493 (1.4)	263 (0.7)	451 (0.9)	227 (0.5)
Any malignancy, including lymphoma and leukaemia, except malignant neoplasm of skin[e]	0 (0.00)	0 (0.00)	0 (0.00)	0 (0.00)	0 (0.00)	0 (0.00)	0 (0.00)	0 (0.00)
Cerebrovascular disease	124 (1.6)	52 (0.7)	885 (1.5)	390 (0.7)	1169 (3.2)	915 (2.5)	1434 (2.9)	807 (1.6)
Chronic pulmonary disease	792 (10.2)	284 (3.7)	6011 (10.3)	2480 (4.3)	7892 (21.8)	4754 (13.1)	13,857 (27.9)	5607 (11.3)
Congestive heart failure	103 (1.3)	28 (0.4)	532 (0.9)	232 (0.4)	1296 (3.6)	1010 (2.8)	1400 (2.8)	816 (1.6)
Dementia	37 (0.5)	2 (0.0)	71 (0.1)	3 (0.0)	562 (1.6)	61 (0.2)	161 (0.3)	41 (0.1)
Diabetes with chronic complication	200 (2.6)	53 (0.7)	952 (1.6)	438 (0.8)	1804 (5.0)	1098 (3.1)	1847 (3.7)	1041 (2.1)
Diabetes without chronic complication	1136 (14.6)	410 (5.3)	5389 (9.3)	3039 (5.2)	7708 (21.2)	4571 (12.6)	7478 (15.0)	4528 (9.1)

Hemiplegia or paraplegia	31 (0.4)	8 (0.1)	168 (0.3)	64 (0.1)	440 (1.2)	476 (1.3)	527 (1.1)	508 (1.0)
Metastatic solid tumour[e]	0 (0.0)	0 (0.0)	0 (0.0)	0 (0.0)	0 (0.0)	0 (0.0)	0 (0.0)	0 (0.0)
Mild liver disease	192 (2.5)	78 (1.0)	1380 (2.4)	604 (1.0)	1545 (4.3)	941 (2.6)	2707 (5.4)	972 (2.0)
Moderate or severe liver disease	5 (0.1)	2 (0.0)	72 (0.1)	19 (0.0)	92 (0.3)	92 (0.3)	161 (0.3)	78 (0.2)
Myocardial infarction	35 (0.5)	16 (0.2)	234 (0.40)	113 (0.2)	578 (1.6)	511 (1.4)	790 (1.6)	385 (0.8)
Peptic ulcer disease	105 (1.4)	54 (0.7)	577 (1.0)	322 (0.6)	240 (0.7)	202 (0.6)	477 (1.0)	180 (0.4)
Peripheral vascular disease	105 (1.4)	54 (0.7)	577 (1.0)	322 (0.6)	1497 (4.1)	821 (2.3)	1138 (2.3)	680 (1.4)
Renal disease	151 (1.9)	41 (0.5)	974 (1.7)	343 (0.6)	1098 (3.0)	742 (2.0)	1002 (2.0)	679 (1.4)
Rheumatic disease	76 (1.0)	47 (0.6)	911 (1.6)	512 (0.9)	363 (1.0)	457 (1.3)	957 (1.9)	625 (1.3)
Non-CCI comorbidities, n (%)[d]								
Pain	2869 (36.9)	2218 (28.5)	29,527 (50.7)	17,943 (30.8)	17,854 (49.2)	15,887 (43.8)	33,820 (68.0)	20,925 (42.1)
Substance use disorders[f]	795 (10.21)	48 (0.6)	5451 (9.4)	382 (0.7)	7937 (21.9)	2217 (6.1)	11,571 (23.3)	2463 (5.0)
Nicotine dependence	746 (9.6)	144 (1.9)	4168 (7.2)	1094 (1.9)	11,024 (30.4)	5711 (15.7)	17,310 (34.8)	6795 (13.7)
Anxiety disorders	1749 (22. 5)	369 (4.7)	17,931 (30.8)	3306 (5.7)	8730 (24.1)	3850 (10.6)	19,620 (39.5)	5259 (10.6)

Depressive disorders	1769 (22.7)	344 (4.4)	15,904 (27.3)	3191 (5.5)	11,685 (32.2)	4417 (12.2)	18,019 (36.2)	5877 (11.8)
Posttraumatic stress disorder	334 (4.3)	29 (0.4)	3364 (5.8)	218 (0.4)	2424 (6.7)	409 (1.1)	6669 (13.4)	660 (1.3)
Attention-deficit/hyperactivity disorder	324 (4.2)	120 (1.5)	6626 (11.4)	930 (1.6)	1168 (3.2)	418 (1.2)	5355 (10.8)	747 (1.5)
Personality disorders	252 (3.2)	3 (0.0)	2482 (4.3)	22 (0.0)	2484 (6.9)	101 (0.3)	3676 (7.4)	134 (0.3)
Medications, n (%)								
Antipsychotics	6072 (78.0)	36 (0.5)	25,570 (43.9)	321 (0.6)	30,084 (82.9)	989 (2.7)	27,044 (54.4)	1098 (2.2)
Antidepressants	3734 (48.0)	875 (11.2)	35,529 (61.1)	7608 (13.1)	19,596 (54.0)	6823 (18.8)	31,820 (64.0)	8965 (18.0)
Mood stabilizers	2627 (33.8)	254 (3.3)	37,352 (64.2)	1855 (3.2)	14,087 (38.8)	4258 (11.7)	28,185 (56.7)	5070 (10.2)
Anticonvulsants	3465 (44.5)	471 (6.1)	38,800 (66.7)	3697 (6.4)	17,290 (47.7)	6084 (16.8)	30,985 (62.3)	7283 (14.6)
Anxiolytics	1833 (23.6)	396 (5.1)	18,330 (31.5)	3421 (5.9)	10,463 (28.8)	4025 (11.1)	20,288 (40.8)	5226 (10.5)
Sedatives and hypnotics	735 (9.4)	227 (2.9)	8686 (14.9)	1708 (2.9)	5552 (15.3)	1519 (4.2)	8332 (16.8)	1901 (3.8)

[a]Race not available in the Commercial database.
[b]Regional division not available in the Medicaid database.
[c]CCI is a weighted score based on the number and the seriousness (scored 1–6) of comorbid diseases; higher scores are associated with a greater risk of mortality [1].
[d]Comorbidities were assessed by ≥1 ICD-9-CM or ICD-10-CM diagnosis code for the particular condition occurring during the baseline period, except for pain (≥2 diagnosis codes).
[e]Individuals with any cancer or metastatic cancer diagnoses during the analytic window were excluded from the analysis.
[f]Not including nicotine dependence.
CCI, Charlson Comorbidity Index.

REFERENCE

1. Charlson ME, Pompei P, Ales KL, MacKenzie CR. A new method of classifying prognostic comorbidity in longitudinal studies: development and validation. J Chronic Dis. 1987;40:373-83. 10.1016/0021-9681(87)90171-8.
