# Supplementary material for: Prescription opioid dispensing patterns among patients with schizophrenia or bipolar disorder
Source: BMC Psychiatry. 2024 Apr 2;24:244. doi: 10.1186/s12888-024-05676-5 (PMC10986122; doi:10.1186/s12888-024-05676-5)
Supplement: Supplementary file 2 — Additional File 2. Demographic and Clinical Characteristics for Patients With Schizophrenia or Bipolar Disorder and Matched Controls: 2016. [file 12888_2024_5676_MOESM2_ESM.pdf]

Additional File 2. Demographic and Clinical Characteristics for Patients With Schizophrenia or Bipolar Disorder and Matched Controls: 2016

	Commercial Database[a] 2016				Medicaid Database[b] 2016			
	**Patients With Schizophrenia (N=7606)**	**Schizophrenia Controls (N=7606)**	**Patients With Bipolar Disorder (N=57,442)**	**Bipolar Disorder Controls (N=57,442)**	**Patients With Schizophrenia (N=39,574)**	**Schizophrenia Controls (N=39,574)**	**Patients With Bipolar Disorder (N=59,952)**	**Bipolar Disorder Controls (N=59,952)**
Age, mean (SD)	41.0 (14.8)	41.0 (14.8)	42.5 (13.6)	42.5 (13.6)	43.9 (12.6)	43.9 (12.6)	38.9 (12.3)	38.9 (12.3)
Median (Q1–Q3)	42 (25–55)	42 (25–55)	44 (31–54)	44 (31–54)	45 (33–55)	45 (33–55)	38 (29–49)	38 (29–49)
Age category, n (%)								
18–30 years	2566 (33.7)	2566 (33.7)	13,896 (24.2)	13,896 (24.)	7586 (19.2)	7586 (19.2)	17,437 (29.1)	17,437 (29.1)
31–45 years	1643 (21.6)	1643 (21.6)	16,725 (29.1)	16,725 (29.1)	12,315 (31.1)	12,315 (31.1)	22,963 (38.3)	22,963 (38.3)
46–60 years	2630 (34.6)	2630 (34.6)	21,812 (38.0)	21,812 (38.0)	16,493 (41.7)	16,493 (41.7)	17,510 (29.2)	17,510 (29.2)
61–64 years	767 (10.1)	767 (10.1)	5009 (8.7)	5009 (8.7)	3180 (8.0)	3180 (8.0)	2042 (3.4)	2042 (3.4)
Sex, n (%)								
Female	3447 (45.3)	3447 (45.3)	37,444 (65.2)	37,444 (65.2)	17,867 (45.2)	17,867 (45.2)	42,606 (71.1)	42,606 (71.1)
Male	4159 (54.7)	4159 (54.7)	19,998 (34.8)	19,998 (34.8)	21,707 (54.9)	21,707 (54.9)	17,346 (28.9)	17,346 (28.9)
Race (Medicaid database only), n (%)								
Black	—	—	—	—	18,467 (46.7)	12,096 (30.6)	10,957 (18.3)	20,015 (33.4)
White	—	—	—	—	15,646 (39.5)	19,362 (48.9)	41,679 (69.5)	29,658 (49.5)
Hispanic	—	—	—	—	631 (1.6)	832 (2.1)	832 (1.4)	1567 (2.6)
Other	—	—	—	—	4830 (12.2)	7284 (18.4)	6484 (10.8)	8712 (14.5)
Regional division (Commercial database only), n (%)								

East North Central	1343 (17.7)	1121 (14.7)	9616 (16.7)	8825 (15.4)	—	—	—	—
East South Central	485 (6.4)	673 (8.9)	4820 (8.4)	5079 (8.8)	—	—	—	—
Middle Atlantic	1634 (21.5)	1003 (13.2)	8080 (14.1)	7417 (12.9)	—	—	—	—
Mountain	386 (5.1)	503 (6.61)	3976 (6.9)	3822 (6.7)	—	—	—	—
New England	262 (3.4)	270 (3.6)	2401 (4.2)	2005 (3.5)	—	—	—	—
Pacific	730 (9.6)	812 (10.9)	5984 (10.4)	5979 (10.4)	—	—	—	—
South Atlantic	1572 (20.7)	1800 (23.7)	12,919 (22.5)	13,493 (23.5)	—	—	—	—
West North Central	378 (5.0)	379 (5.0)	2815 (4.9)	2777 (4.8)	—	—	—	—
West South Central	776 (10.2)	1008 (13.3)	6625 (11.5)	7790 (13.6)	—	—	—	—
Unknown	40 (0.5)	37 (0.5)	206 (0.4)	255 (0.4)	—	—	—	—
CCI[c,d]								
Mean (SD)	0.28 (0.7)	0.11 (0.4)	0.25 (0.7)	0.11 (0.4)	0.61 (1.2)	0.32 (0.8)	0.59 (1.1)	0.23 (0.7)
Median (Q1–Q3)	0 (0–0)	0 (0–0)	0 (0–0)	0 (0–0)	0 (0–1)	0 (0–0)	0 (0–1)	0 (0–0)
Individual comorbidities included in the CCI, n (%)								
AIDS/HIV	24 (0.3)	12 (0.2)	179 (0.3)	64 (0.1)	549 (1.4)	237 (0.6)	546 (0.9)	232 (0.4)
Any malignancy, including lymphoma and leukaemia, except malignant neoplasm of skin[e]	0 (0.00)	0 (0.0)	0 (0.0)	0 (0.0)	1 (0.0)	0 (0.0)	0 (0.0)	0 (0.0)
Cerebrovascular disease	125 (1.6)	54 (0.7)	912 (1.6)	363 (0.6)	1163 (2.9)	835 (2.1)	1546 (2.6)	818 (1.4)
Chronic pulmonary disease	748 (9.8)	321 (4.2)	5866 (10.2)	2537 (4.4)	8678 (21.9)	4366 (11.0)	16,212 (27.0)	5625 (9.4)

Congestive heart failure	99 (1.3)	41 (0.5)	558 (1.0)	250 (0.4)	1483 (3.8)	942 (2.4)	1656 (2.8)	882 (1.5)
Dementia	48 (0.6)	2 (0.0)	170 (0.3)	8 (0.0)	813 (2.1)	83 (0.2)	289 (0.5)	64 (0.1)
Diabetes with chronic complication	257 (3.4)	78 (1.0)	1230 (2.1)	529 (0.9)	2253 (5.7)	1222 (3.1)	2489 (4.2)	1266 (2.1)
Diabetes without chronic complication	1076 (14.2)	360 (4.7)	5442 (9.5)	3182 (5.5)	8458 (21.4)	4131 (10.4)	8811 (14.7)	4605 (7.7)
Hemiplegia or paraplegia	41 (0.5)	9 (0.1)	146 (0.3)	65 (0.1)	401 (1.0)	388 (1.0)	560 (0.9)	440 (0.7)
Metastatic solid tumour[e]	0 (0.0)	0 (0.0)	0 (0.0)	0 (0.0)	0 (0.0)	0 (0.0)	0 (0.0)	0 (0.0)
Mild liver disease	207 (2.7)	67 (1.0)	1381 (2.4)	646 (1.1)	1835 (4.6)	963 (2.4)	3431 (5.7)	1029 (1.7)
Moderate or severe liver disease	7 (0.1)	4 (0.1)	61 (0.1)	20 (0.0)	125 (0.3)	92 (0.2)	189 (0.3)	57 (0.1)
Myocardial infarction	38 (0.5)	25 (0.3)	242 (0.4)	111 (0.2)	646 (1.6)	481 (1.2)	935 (1.6)	439 (0.7)
Peptic ulcer disease	66 (0.9)	33 (0.4)	472 (0.8)	205 (0.4)	272 (0.7)	178 (0.5)	557 (0.9)	215 (0.4)
Peripheral vascular disease	94 (1.2)	55 (0.7)	658 (1.2)	313 (0.5)	1595 (4.0)	831 (2.1)	1413 (2.5)	750 (1.3)
Renal disease	146 (1.9)	52 (0.7)	1085 (1.9)	365 (0.6)	1245 (3.2)	724 (1.8)	1220 (2.0)	680 (1.1)
Rheumatic disease	65 (0.8)	53 (0.7)	1007 (1.8)	507 (0.9)	433 (1.1)	417 (1.1)	1119 (1.9)	595 (1.0)
Non-CCI comorbidities, n (%)[d]								
Pain	2823 (37.1)	2179 (28.7)	29,225 (50.9)	17,503 (30.5)	19,896 (50.3)	15,125 (38.2)	40,584 (67.7)	21,889 (36.5)
Substance use disorders[f]	803 (10.6)	55 (0.7)	5799 (10.1)	391 (0.7)	9117 (23.0)	2419 (6.1)	14,832 (24.7)	2757 (4.6)
Nicotine dependence	686 (9.0)	138 (1.8)	4311 (7.5)	954 (1.7)	12,480 (31.5)	5715 (14.4)	21,462 (35.8)	7463 (12.5)
Anxiety disorders	1935 (25.4)	382 (5.0)	19,753 (34.4)	3464 (6.0)	10,143 (25.6)	3952 (10.0)	24,650 (41.1)	5988 (10.0)

Depressive disorders	1850 (24.3)	340 (4.5)	16,178 (28.7)	2976 (5.2)	12,264 (31.0)	4216 (10.7)	21,227 (35.4)	6214 (10.4)
Posttraumatic stress disorder	377 (5.0)	15 (0.2)	3776 (6.6)	220 (0.4)	3030 (7.7)	451 (1.1)	8874 (14.8)	712 (1.2)
Attention-deficit/hyperactivity disorder	395 (5.2)	149 (2.0)	7847 (13.7)	1011 (1.8)	1388 (3.5)	484 (1.2)	6874 (11.5)	842 (1.4)
Personality disorders	290 (3.8)	7 (0.1)	2671 (4.7)	21 (0.0)	3002 (7.6)	120 (0.3)	4782 (8.0)	149 (0.3)
Medications, n (%)								
Antipsychotics	6003 (78.9)	29 (0.4)	26,325 (45.8)	339 (0.6)	32,620 (82.4)	912 (2.3)	32,042 (53.5)	1235 (2.1)
Antidepressants	3697 (48.6)	882 (11.6)	35,600 (62.0)	7510 (13.1)	21,470 (54.3)	6858 (17.3)	37,789 (63.0)	10,002 (16.7)
Mood stabilizers	2593 (34.1)	207 (2.7)	37,172 (64.7)	1892 (3.3)	15,846 (40.0)	4367 (11.0)	33,614 (56.1)	5635 (9.4)
Anticonvulsants	3318 (43.6)	399 (5.3)	38,490 (67.0)	3657 (6.4)	18,967 (48.0)	6050 (15.3)	36,921 (61.6)	7945 (13.3)
Anxiolytics	1810 (23.8)	363 (4.8)	18,497 (32.2)	3423 (6.0)	11,663 (29.5)	3907 (9.9)	24,523 (40.9)	5557 (9.3)
Sedatives and hypnotics	658 (8.7)	194 (2.6)	8500 (14.8)	1599 (2.8)	5669 (14.3)	1396 (3.5)	9286 (15.5)	1904 (3.2)

[a]Race not available in the Commercial database.
[b]Regional division not available in the Medicaid database.
[c]CCI is a weighted score based on the number and the seriousness (scored 1–6) of comorbid diseases; higher scores are associated with a greater risk of mortality [1].
[d]Comorbidities were assessed by ≥1 ICD-9-CM or ICD-10-CM diagnosis code for the particular condition occurring during the baseline period, except for pain (≥2 diagnosis codes).
[e]Individuals with any cancer or metastatic cancer diagnoses during the analytic window were excluded from the analysis.
[f]Not including nicotine dependence.
CCI, Charlson Comorbidity Index.

REFERENCE

1. Charlson ME, Pompei P, Ales KL, MacKenzie CR. A new method of classifying prognostic comorbidity in longitudinal studies: development and validation. J Chronic Dis. 1987;40:373-83. 10.1016/0021-9681(87)90171-8.
