# Supplementary material for: Prescription opioid dispensing patterns among patients with schizophrenia or bipolar disorder
Source: BMC Psychiatry. 2024 Apr 2;24:244. doi: 10.1186/s12888-024-05676-5 (PMC10986122; doi:10.1186/s12888-024-05676-5)
Supplement: Supplementary file 3 — Additional File 3. Demographic and Clinical Characteristics for Patients With Schizophrenia or Bipolar Disorder and Matched Controls: 2017. [file 12888_2024_5676_MOESM3_ESM.pdf]

Additional File 3. Demographic and Clinical Characteristics for Patients With Schizophrenia or Bipolar Disorder and Matched Controls: 2017

	Commercial Database[a] 2017				Medicaid Database[b] 2017			
	**Patients With Schizophrenia (N=5182)**	**Schizophrenia Controls (N=5182)**	**Patients With Bipolar Disorder (N=49,907)**	**Bipolar Disorder Controls (N=49,907)**	**Patients With Schizophrenia (N=34,972)**	**Schizophrenia Controls (N=34,972)**	**Patients With Bipolar Disorder (N=69,987)**	**Bipolar Disorder Controls (N=69,987)**
Age, mean (SD)	40.2 (14.8)	40.2 (14.8)	42.3 (13.7)	42.3 (13.7)	43.6 (12.8)	43.6 (12.8)	38.9 (12.3)	38.9 (12.3)
Median (Q1–Q3)	40 (25–54)	40 (25–54)	44 (31–5)	44 (31–54)	44 (33–55)	44 (33–55)	38 (29–49)	38 (29–49)
Age category, n (%)								
18–30 years	1860 (35.9)	1860 (35.9)	12,336 (24.7)	12,336 (24.7)	7074 (20.2)	7074 (20.2)	20,135 (28.8)	20,135 (28.8)
31–45 years	1193 (23.0)	1193 (23.0)	14,501 (29.1)	14,501 (29.1)	11,090 (31.7)	11,090 (31.1)	27,302 (39.0)	27,302 (39.0)
46–60 years	1609 (31.1)	1609 (31.1)	18,814 (37.7)	18,814 (37.7)	13,764 (39.4)	13,764 (39.4)	20,128 (28.8)	20,128 (28.8)
61–64 years	520 (10.0)	520 (10.0)	4256 (8.5)	4256 (8.5)	3044 (8.7)	3044 (8.7)	2422 (3.5)	2422 (3.5)
Sex, n (%)								
Female	2210 (42.7)	2210 (42.7)	32,721 (65.6)	32,721 (65.6)	14,552 (41.6)	14,552 (41.6)	49,532 (70.8)	49,532 (70.8)
Male	2972 (57.4)	2972 (57.4)	17,186 (34.4)	17,186 (34.4)	20,420 (58.4)	20,420 (58.4)	20,455 (29.2)	20,455 (29.2)
Race (Medicaid database only), n (%)								
Black	—	—	—	—	16,890 (48.3)	11,176 (32.0)	13,313 (19.0)	24,648 (35.2)
White	—	—	—	—	13,346 (38.2)	16,836 (48.1)	48,317 (69.0)	33,602 (48.0)
Hispanic	—	—	—	—	569 (1.6)	782 (2.2)	1032 (1.5)	1976 (2.8)
Other	—	—	—	—	4167 (11.9)	6178 (17.7)	7325 (10.)	9761 (14.0)

Regional division (Commercial database only), n (%)								
East North Central	808 (15.6)	731 (14.1)	7734 (15.5)	7123 (14.3)	—	—	—	—
East South Central	298 (5.8)	351 (6.8)	3306 (6.6)	3334 (6.7)	—	—	—	—
Middle Atlantic	767 (14.8)	492 (9.49)	4822 (9.7)	4580 (9.2)	—	—	—	—
Mountain	189 (3.65)	233 (4.50)	2433 (4.9)	2371 (4.8)	—	—	—	—
New England	177 (3.42)	164 (3.16)	2161 (4.3)	1792 (3.6)	—	—	—	—
Pacific	469 (9.05)	564 (10.88)	4578 (9.2)	4965 (10.0)	—	—	—	—
South Atlantic	909 (17.5)	1058 (20.4)	9687 (19.4)	10,451 (20.9)	—	—	—	—
West North Central	257 (5.0)	267 (5.2)	2630 (5.3)	2426 (4.9)	—	—	—	—
West South Central	374 (7.2)	504 (9.7)	4332 (8.7)	4997 (10.0)	—	—	—	—
Unknown	934 (18.0)	818 (15.8)	8224 (16.5)	7868 (15.8)	—	—	—	—
CCI[c,d]								
Mean (SD)	0.25 (0.7)	0.09 (0.4)	0.25 (0.7)	0.10 (0.4)	0.60 (1.2)	0.30 (0.8)	0.57 (1.0)	0.22 (0.7)
Median (Q1–Q3)	0 (0–0)	0 (0–0)	0 (0–0)	0 (0–0)	0 (0–1)	0 (0–0)	0 (0–1)	0 (0–0)
Individual comorbidities included in the CCI, n (%)								
AIDS/HIV	18 (0.4)	5 (0.1)	155 (0.3)	61 (0.1)	530 (1.5)	206 (0.6)	640 (0.9)	251 (0.4)
Any malignancy, including lymphoma and leukaemia, except malignant neoplasm of skin[e]	0 (0.0)	0 (0.0)	0 (0.0)	0 (0.0)	0 (0.0)	0 (0.00)	0 (0.00)	0 (0.0)
Cerebrovascular disease	83 (1.6)	28 (0.5)	746 (1.5)	247 (0.5)	986 (2.8)	675 (1.9)	1618 (2.3)	809 (1.2)

Chronic pulmonary disease	476 (9.2)	219 (4.2)	5076 (10.2)	2088 (4.2)	7187 (20.6)	3543 (10.1)	17,847 (25.5)	6123 (8.8)
Congestive heart failure	66 (1.3)	25 (0.5)	448 (0.9)	199 (0.4)	1262 (3.6)	810 (2.3)	1830 (2.6)	943 (1.4)
Dementia	28 (0.5)	3 (0.1)	152 (0.3)	10 (0.0)	879 (2.5)	75 (0.2)	380 (0.5)	93 (0.1)
Diabetes with chronic complication	171 (3.3)	49 (1.0)	1142 (2.3)	483 (1.0)	2064 (5.9)	1075 (3.1)	3206 (4.6)	1445 (2.1)
Diabetes without chronic complication	731 (14.1)	229 (4.4)	4757 (9.5)	2706 (5.4)	7090 (20.3)	3285 (9.4)	9963 (14.2)	4893 (7.0)
Hemiplegia or paraplegia	22 (0.4)	5 (0.1)	122 (0.2)	45 (0.1)	290 (0.8)	265 (0.8)	428 (0.6)	312 (0.5)
Metastatic solid tumour[e]	0 (0.0)	0 (0.0)	0 (0.0)	0 (0.0)	0 (0.0)	0 (0.0)	0 (0.0)	0 (0.0)
Mild liver disease	135 (2.6)	59 (1.1)	1297 (2.6)	475 (1.0)	1521 (4.4)	844 (2.4)	3973 (5.7)	1209 (1.7)
Moderate or severe liver disease	3 (0.1)	2 (0.0)	71 (0.1)	13 (0.0)	103 (0.3)	83 (0.2)	205 (0.3)	89 (0.1)
Myocardial infarction	31 (0.6)	24 (0.5)	218 (0.4)	103 (0.2)	521 (1.5)	408 (1.2)	1043 (1.5)	486 (0.7)
Peptic ulcer disease	13 (0.3)	6 (0.1)	267 (0.5)	79 (0.2)	239 (0.7)	130 (0.4)	577 (0.8)	224 (0.3)
Peripheral vascular disease	87 (1.7)	30 (0.6)	576 (1.2)	274 (0.6)	1367 (3.9)	726 (2.1)	1609 (2.3)	831 (1.2)
Renal disease	110 (2.1)	40 (0.8)	913 (1.8)	314 (0.6)	1176 (3.4)	667 (1.9)	1513 (2.2)	774 (1.1)
Rheumatic disease	35 (0.7)	41 (0.8)	835 (1.7)	423 (0.9)	337 (1.0)	319 (0.9)	1190 (1.7)	625 (0.9)
Non-CCI comorbidities, n (%)[d]								
Pain	1835 (35.4)	1405 (27.1)	25,179 (50.5)	15,059 (30.2)	16,889 (48.3)	12,300 (35.2)	45,960 (65.7)	23,432 (33.5)
Substance use disorders[f]	572 (11.0)	39 (0.8)	4964 (10.0)	329 (0.7)	7387 (21.1)	2085 (6.0)	16,775 (24.0)	3238 (4.6)
Nicotine dependence	528 (10.2)	89 (1.7)	3870 (7.8)	764 (1.5)	10,495 (30.0)	4724 (13.5)	23,896 (34.1)	8163 (11.7)

Anxiety disorders	1274 (24.6)	281 (5.4)	17,526 (35.1)	3,156 (6.3)	8,503 (24.3)	3220 (9.2)	28,256 (40.4)	6585 (9.4)
Depressive disorders	1148 (22.2)	233 (4.5)	12,997 (26.0)	2527 (5.1)	8497 (24.3)	3103 (8.9)	21,778 (31.1)	6204 (8.9)
Posttraumatic stress disorder	246 (4.8)	13 (0.3)	3540 (7.1)	187 (0.4)	2606 (7.5)	380 (1.1)	11,068 (15.8)	886 (1.3)
Attention-deficit/hyperactivity disorder	263 (5.1)	106 (2.1)	6875 (13.8)	843 (1.7)	1158 (3.3)	440 (1.3)	7993 (11.4)	1038 (1.5)
Personality disorders	172 (3.3)	4 (0.1)	1806 (3.6)	25 (0.1)	2243 (6.4)	84 (0.2)	4922 (7.0)	153 (0.2)
Medications, n (%)								
Antipsychotics	4083 (78.89)	37 (0.7)	23,978 (48.1)	328 (0.7)	28,554 (81.7)	783 (2.2)	38,080 (54.4)	1338 (1.9)
Antidepressants	2381 (46.0)	561 (10.8)	30,907 (61.9)	6810 (13.7)	17,839 (51.0)	5536 (15.8)	42,942 (61.4)	10,536 (15.1)
Mood stabilizers	1598 (30.8)	147 (2.8)	32,814 (65.8)	1790 (3.6)	13,193 (37.7)	3731 (10.7)	39,024 (55.8)	6185 (8.8)
Anticonvulsants	2106 (40.6)	296 (5.7)	33,871 (67.9)	3218 (6.5)	15,850 (45.3)	4974 (14.2)	42,291 (60.4)	8374 (12.0)
Anxiolytics	1,204 (23.2)	249 (4.8)	16,157 (32.4)	2858 (5.7)	9467 (27.1)	2982 (8.5)	27,774 (39.7)	5663 (8.1)
Sedatives and hypnotics	395 (7.6)	119 (2.3)	6823 (13.7)	1273 (2.6)	4276 (12.2)	1053 (3.0)	9653 (13.8)	1773 (2.5)

[a]Race not available in the Commercial database.
[b]Regional division not available in the Medicaid database.
[c]CCI is a weighted score based on the number and the seriousness (scored 1–6) of comorbid diseases; higher scores are associated with a greater risk of mortality [1].
[d]Comorbidities were assessed by ≥1 ICD-9-CM or ICD-10-CM diagnosis code for the particular condition occurring during the baseline period, except for pain (≥2 diagnosis codes).
[e]Individuals with any cancer or metastatic cancer diagnoses during the analytic window were excluded from the analysis.
[f]Not including nicotine dependence.
CCI, Charlson Comorbidity Index.

REFERENCE

1. Charlson ME, Pompei P, Ales KL, MacKenzie CR. A new method of classifying prognostic comorbidity in longitudinal studies: development and validation. J Chronic Dis. 1987;40:373-83. 10.1016/0021-9681(87)90171-8.
