# Supplementary material for: Prescription opioid dispensing patterns among patients with schizophrenia or bipolar disorder
Source: BMC Psychiatry. 2024 Apr 2;24:244. doi: 10.1186/s12888-024-05676-5 (PMC10986122; doi:10.1186/s12888-024-05676-5)
Supplement: Supplementary file 4 — Additional File 4. Demographic and Clinical Characteristics for Patients With Schizophrenia or Bipolar Disorder and Matched Controls: 2018. [file 12888_2024_5676_MOESM4_ESM.pdf]

Additional File 4. Demographic and Clinical Characteristics for Patients With Schizophrenia or Bipolar Disorder and Matched Controls: 2018

	Commercial Database[a] 2018				Medicaid Database[b] 2018			
	**Patients With Schizophrenia (N=4732)**	**Schizophrenia Controls (N=4732)**	**Patients With Bipolar Disorder (N=50,079)**	**Bipolar Disorder Controls (N=50,079)**	**Patients With Schizophrenia (N=29,967)**	**Schizophrenia Controls (N=29,967)**	**Patients With Bipolar Disorder (N=62,341)**	**Bipolar Disorder Controls (N=62,341)**
Age, mean (SD)	39.2 (14.7)	39.2 (14.7)	41.8 (13.7)	41.8 (13.7)	43.6 (12.8)	43.6 (12.8)	39.2 (12.4)	39.2 (12.4)
Median (Q1–Q3)	37 (25–53)	37 (25–53)	43 (30–54)	43 (30–54)	44 (33–55)	44 (33–55)	38 (29–49)	38 (29–49)
Age category, n (%)								
18–30 years	1851 (39.1)	1851 (39.1)	13,009 (26.0)	13,009 (26.0)	6117 (20.4)	6117 (20.4)	17,569 (28.2)	17,569 (28.2)
31–45 years	1061 (22.4)	1061 (22.4)	14,565 (29.1)	14,565 (29.1)	9619 (32.1)	9619 (32.1)	23,937 (38.4)	23,937 (38.4)
46–60 years	1403 (29.7)	1403 (29.7)	18,455 (36.9)	18,455 (36.9)	11,471 (38.3)	11,471 (38.3)	18,438 (29.6)	18,438 (29.6)
61–64 years	417 (8.8)	417 (8.8)	4050 (8.1)	4050 (8.1)	2760 (9.2)	2760 (9.2)	2397 (3.8)	2397 (3.8)
Sex, n (%)								
Female	1981 (41.9)	1981 (41.9)	32,767 (65.4)	32,767 (65.4)	12,209 (40.7)	12,209 (40.7)	44,104 (70.8)	44,104 (70.8)
Male	2751 (58.1)	2751 (58.1)	17,312 (34.6)	17,312 (34.6)	17,758 (59.3)	17,758 (59.3)	18,237 (29.3)	18,237 (29.3)
Race (Medicaid database only), n (%)								
Black	—	—	—	—	14,466 (48.3)	9973 (33.3)	12,536 (20.1)	22,549 (36.2)
White	—	—	—	—	11,447 (38.2)	14,833 (49.5)	42,852 (68.7)	30,612 (49.1)
Hispanic	—	—	—	—	511 (1.7)	687 (2.3)	865 (1.4)	1809 (2.9)
Other	—	—	—	—	3439 (11.5)	4132 (13.8)	5915 (9.5)	6888 (11.1)
Unknown	—	—	—	—	104 (0.4)	342 (1.1)	173 (0.3)	483 (0.8)

Regional division (Commercial database only), n (%)								
East North Central	842 (17.8)	785 (16.6)	9063 (18.1)	8367 (16.7)	—	—	—	—
East South Central	274 (5.8)	292 (6.2)	3173 (6.3)	3111 (6.2)	—	—	—	—
Middle Atlantic	960 (20.3)	670 (14.2)	7856 (15.7)	7240 (14.5)	—	—	—	—
Mountain	163 (3.4)	216 (4.6)	2356 (4.7)	2301 (4.6)	—	—	—	—
New England	153 (3.2)	145 (3.1)	2065 (4.1)	1735 (3.5)	—	—	—	—
Pacific	471 (10.0)	498 (10.5)	4565 (9.1)	5257 (10.5)	—	—	—	—
South Atlantic	807 (17.1)	910 (19.2)	9079 (18.1)	9411 (18.8)	—	—	—	—
West North Central	251 (5.3)	267 (5.6)	2747 (5.5)	2597 (5.2)	—	—	—	—
West South Central	352 (7.4)	464 (9.8)	3998 (8.0)	4634 (9.3)	—	—	—	—
Unknown	459 (9.7)	485 (10.3)	5177 (10.3)	5426 (10.8)	—	—	—	—
CCI[c,d]								
Mean (SD)	0.26 (0.7)	0.10 (0.4)	0.25 (0.7)	0.10 (0.4)	0.63 (1.2)	0.31 (0.8)	0.60 (1.1)	0.23 (0.0)
Median (Q1–Q3)	0 (0–0)	0 (0–0)	0 (0–0)	0 (0–0)	0 (0–1)	0 (0–0)	0 (0–1)	0 (0–0)
Individual comorbidities included in the CCI, n (%)								
AIDS/HIV	17 (0.4)	5 (0.1)	183 (0.4)	62 (0.1)	496 (1.7)	190 (0.6)	619 (1.0)	202 (0.3)
Any malignancy, including lymphoma and leukaemia, except malignant neoplasm of skin[e]	0 (0.0)	0 (0.0)	0 (0.0)	0 (0.0)	0 (0.0)	1 (0.0)	0 (0.0)	0 (0.0)
Cerebrovascular disease	80 (1.7)	35 (0.7)	652 (1.3)	286 (0.6)	882 (2.9)	575 (1.9)	1431 (2.3)	749 (1.2)

Chronic pulmonary disease	395 (8.4)	195 (4.1)	5000 (10.0)	2100 (4.2)	6306 (21.0)	3048 (10.2)	16,104 (25.8)	5523 (8.9)
Congestive heart failure	53 (1.1)	19 (0.4)	431 (0.9)	208 (0.4)	1140 (3.8)	733 (2.5)	1748 (2.8)	951 (1.5)
Dementia	40 (0.9)	1 (0.0)	107 (0.2)	12 (0.0)	793 (2.7)	76 (0.3)	339 (0.5)	76 (0.1)
Diabetes with chronic complication	155 (3.3)	58 (1.2)	1,181 (2.4)	519 (1.0)	2001 (6.7)	1009 (3.4)	3195 (5.1)	1554 (2.5)
Diabetes without chronic complication	575 (12.2)	234 (5.0)	4617 (9.2)	2566 (5.1)	6100 (20.4)	2865 (9.6)	8935 (14.3)	4413 (7.1)
Hemiplegia or paraplegia	17 (0.4)	3 (0.1)	105 (0.2)	29 (0.1)	248 (0.8)	233 (0.8)	438 (0.7)	288 (0.5)
Metastatic solid tumour[e]	0 (0.00)	0 (0.0)	0 (0.0)	0 (0.0)	0 (0.0)	0 (0.0)	0 (0.0)	0 (0.0)
Mild liver disease	120 (2.5)	43 (0.9)	1259 (2.5)	511 (1.0)	1443 (4.8)	762 (2.5)	3880 (6.2)	1152 (1.9)
Moderate or severe liver disease	5 (0.1)	0 (0.0)	46 (0.1)	11 (0.0)	86 (0.3)	59 (0.2)	200 (0.3)	85 (0.1)
Myocardial infarction	18 (0.4)	5 (0.1)	220 (0.4)	115 (0.2)	476 (1.6)	352 (1.2)	923 (1.5)	396 (0.6)
Peptic ulcer disease	16 (0.3)	7 (0.2)	238 (0.5)	55 (0.1)	191 (0.6)	104 (0.4)	546 (0.9)	174 (0.3)
Peripheral vascular disease	68 (1.4)	33 (0.7)	551 (1.1)	284 (0.6)	1292 (4.3)	636 (2.1)	1473 (2.4)	751 (1.2)
Renal disease	86 (1.8)	32 (0.7)	936 (1.9)	304 (0.6)	1015 (3.4)	560 (1.9)	1474 (2.4)	733 (1.2)
Rheumatic disease	35 (0.7)	29 (0.6)	827 (1. 7)	406 (0.8)	258 (0.9)	255 (0.9)	1053 (1.7)	569 (0.9)
Non-CCI comorbidities, n (%)[d]								
Pain	1664 (35.2)	1261 (26.7)	24,983 (49.9)	15,335 (30.6)	14,431 (48.2)	10,260 (34.2)	40,306 (64.7)	20,546 (33.0)
Substance use disorders[f]	573 (12.1)	49 (1.0)	4992 (10.0)	320 (0.6)	6944 (23.2)	1937 (6.5)	15,848 (25.4)	3078 (4.9)
Nicotine dependence	510 (10.8)	70 (1.5)	4070 (8.1)	828 (1.7)	9440 (31.5)	4004 (13.4)	21,607 (34.7)	6956 (11.2)

Anxiety disorders	1264 (26.7)	253 (5.4)	19,180 (38.3)	3544 (7.1)	7828 (26.1)	2863 (9.6)	26,119 (41.9)	6069 (9.7)
Depressive disorders	1102 (23.3)	221 (4.7)	13,289 (26.5)	2755 (5.5)	7679 (25.6)	2763 (9.2)	19,602 (31.4)	5860 (9.4)
Posttraumatic stress disorder	254 (5.4)	12 (0.3)	4002 (8.0)	215 (0.4)	2555 (8.5)	364 (1.2)	11,099 (17.8)	885 (1.4)
Attention-deficit/hyperactivity disorder	297 (6.3)	89 (1.9)	6982 (13.9)	821 (1.6)	1172 (3.9)	333 (1.1)	7126 (11.4)	879 (1.4)
Personality disorders	153 (3.2)	1 (0.02)	1922 (3.8)	21 (0.04)	1899 (6.3)	62 (0.2)	4418 (7.1)	128 (0.2)
Medications, n (%)								
Antipsychotics	3684 (77.9)	33 (0.7)	23,906 (47.7)	304 (0.61)	24,602 (82.1)	707 (2.4)	34,710 (55.7)	1281 (2.1)
Antidepressants	2232 (47.2)	520 (11.0)	30,858 (61.6)	6910 (13.8)	15,470 (51.6)	4974 (16.6)	38,869 (62.4)	9735 (15.6)
Mood stabilizers	1474 (31.2)	160 (3.4)	32,857 (65.6)	1783 (3.6)	11,516 (38.4)	3263 (10.9)	35,032 (56.2)	5822 (9.3)
Anticonvulsants	1910 (40.4)	258 (5.5)	33,686 (67.3)	3182 (6.4)	13,564 (45.3)	4271 (14.3)	37,575 (60.3)	7610 (12.2)
Anxiolytics	1085 (22.9)	213 (4.5)	15,998 (32.0)	2828 (5.7)	8251 (27.5)	2514 (8.4)	25,046 (40.2)	5019 (8.1)
Sedatives and hypnotics	356 (7.5)	85 (1.8)	6230 (12.4)	1146 (2.3)	2815 (9.4)	749 (2.5)	7369 (11.8)	1365 (2.2)

[a]Race not available in the Commercial database.
[b]Regional division not available in the Medicaid database.
[c]CCI is a weighted score based on the number and the seriousness (scored 1–6) of comorbid diseases; higher scores are associated with a greater risk of mortality [1].
[d]Comorbidities were assessed by ≥1 ICD-9-CM or ICD-10-CM diagnosis code for the particular condition occurring during the baseline period, except for pain (≥2 diagnosis codes).
[e]Individuals with any cancer or metastatic cancer diagnoses during the analytic window were excluded from the analysis.
[f]Not including nicotine dependence.
CCI, Charlson Comorbidity Index.

REFERENCE

1. Charlson ME, Pompei P, Ales KL, MacKenzie CR. A new method of classifying prognostic comorbidity in longitudinal studies: development and validation. J Chronic Dis. 1987;40:373-83. 10.1016/0021-9681(87)90171-8.
