# Supplementary material for: Prescription opioid dispensing patterns among patients with schizophrenia or bipolar disorder
Source: BMC Psychiatry. 2024 Apr 2;24:244. doi: 10.1186/s12888-024-05676-5 (PMC10986122; doi:10.1186/s12888-024-05676-5)
Supplement: Supplementary file 5 — Additional File 5. Demographic and Clinical Characteristics for Patients With Schizophrenia or Bipolar Disorder and Matched Controls: 2019. [file 12888_2024_5676_MOESM5_ESM.pdf]

Additional File 5. Demographic and Clinical Characteristics for Patients With Schizophrenia or Bipolar Disorder and Matched Controls: 2019

Parameter	Commercial Database[a] 2019				Medicaid Database[b] 2019			
	Patients With Schizophrenia (N=4773)	Schizophrenia Controls (N=4773)	Patients With Bipolar Disorder (N=52,780)	Bipolar Disorder Controls (N=52,780)	Patients With Schizophrenia (N=30,179)	Schizophrenia Controls (N=30,179)	Patients With Bipolar Disorder (N=63,455)	Bipolar Disorder Controls (N=63,455)
Age, mean (SD)	39.0 (14.7)	39.0 (14.7)	41.5 (13.6)	41.5 (13.6)	43.4 (12.8)	43.4 (12.8)	39.5 (12.4)	39.5 (12.4)
Median (Q1–Q3)	37 (25–53)	37 (25–53)	43 (29–53)	43 (29–53)	43 (32–55)	43 (32–55)	39 (30–50)	39 (30–50)
Age category, n (%)								
18–30 years	1887 (39.5)	1887 (39.5)	14,056 (26.6)	14,056 (26.6)	6170 (20.4)	6170 (20.4)	17,463 (27.5)	17,463 (27.5)
31–45 years	1086 (22.8)	1086 (22.8)	15,661 (29.7)	15,661 (29.7)	10,058 (33.3)	10,058 (33.3)	24,480 (38.6)	24,480 (38.6)
46–60 years	1380 (28.9)	1380 (28.9)	18,984 (36.0)	18,984 (36.0)	11,149 (36.9)	11,149 (36.9)	18,911 (29.8)	18,911 (29.8)
61–64 years	420 (8.8)	420 (8.8)	4079 (7.7)	4079 (7.7)	2802 (9.3)	2802 (9.3)	2601 (4.1)	2601 (4.1)
Sex, n (%)								
Female	1998 (41.9)	1998 (41.9)	34,683 (65.7)	34,683 (65.7)	12,135 (40.2)	12,135 (40.2)	44,925 (70.8)	44,925 (70.8)
Male	2775 (58.1)	2775 (58.1)	18,097 (34.3)	18,097 (34.3)	18,044 (59.8)	18,044 (59.8)	18,530 (29.2)	18,530 (29.2)
Race (Medicaid database only), n (%)								
Black	—	—	—	—	14,569 (48.3)	10,191 (33.8)	12,646 (19.9)	23,223 (36.6)

White	—	—	—	—	11,330 (37.5)	14,840 (49.2)	43,239 (68.1)	30,871 (48.7)
Hispanic	—	—	—	—	539 (1.8)	797 (2.6)	940 (1.5)	1927 (3.0)
Other	—	—	—	—	3295 (10.9)	3564 (11.8)	5875 (9.7)	6345 (10.0)
Unknown	—	—	—	—	446 (1.5)	787 (2.6)	755 (1.2)	1089 (1.7)
Regional division (Commercial database only), n (%)								
East North Central	844 (17.7)	761 (15.9)	9544 (18.1)	8696 (16.5)	—	—	—	—
East South Central	254 (5.3)	245 (5.1)	3273 (6.2)	2863 (5.42)	—	—	—	—
Middle Atlantic	1047 (21.9)	672 (14.1)	8325 (15.8)	7762 (14.7)	—	—	—	—
Mountain	190 (4.0)	242 (5.1)	2724 (5.2)	2629 (5.0)	—	—	—	—
New England	165 (3.5)	153 (3.2)	2188 (4.2)	1780 (3.4)	—	—	—	—
Pacific	345 (7.2)	431 (9.0)	3571 (6.8)	4392 (8.3)	—	—	—	—
South Atlantic	890 (18.7)	942 (19.7)	10,002 (19.0)	10,575 (20.0)	—	—	—	—
West North Central	253 (5.3)	261 (5.5)	2721 (5.2)	2713 (5.1)	—	—	—	—
West South Central	331 (6.9)	551 (11.5)	4757 (9.0)	5668 (10.7)	—	—	—	—
Unknown	454 (9.5)	515 (10.8)	5675 (10.8)	5702 (10.8)	—	—	—	—
CCI[c,d]								

Mean (SD)	0.25 (0.7)	0.09 (0.4)	0.25 (0.7)	0.10 (0.4)	0.64 (1.2)	0.31 (0.9)	0.62 (1.1)	0.23 (0.7)
Median (Q1–Q3)	0 (0–0)	0 (0–0)	0 (0–0)	0 (0–0)	0 (0–1)	0 (0–0)	0 (0–1)	0 (0–0)
Individual comorbidities included in the CCI, n (%)								
AIDS/HIV	16 (0.3)	5 (0.1)	213 (0.4)	80 (0.2)	493 (1.6)	184 (0.6)	634 (1.0)	251 (0.4)
Any malignancy, including lymphoma and leukaemia, except malignant neoplasm of skin[e]	0 (0.0)	0 (0.0)	0 (0.0)	0 (0.0)	0 (0.0)	0 (0.0)	0 (0.0)	0 (0.0)
Cerebrovascular disease	62 (1.3)	25 (0.5)	720 (1.4)	284 (0.5)	1000 (3.3)	561 (1.9)	1626 (2.6)	759 (1.2)
Chronic pulmonary disease	429 (9.0)	152 (3.2)	5184 (9.8)	2166 (4.1)	6303 (20.9)	2966 (9.8)	16,273 (25.6)	5465 (8.6)
Congestive heart failure	65 (1.4)	26 (0.5)	476 (0.9)	210 (0.4)	1152 (3.8)	764 (2.5)	1804 (2.8)	966 (1.5)
Dementia	29 (0.6)	1 (0.0)	126 (0.2)	17 (0.03)	792 (2.6)	68 (0.2)	359 (0.6)	65 (0.1)
Diabetes with chronic complication	137 (2.9)	33 (0.7)	1159 (2.2)	529 (1.0)	1992 (6.6)	1064 (3.5)	3491 (5.5)	1563 (2.5)
Diabetes without chronic complication	595 (12.5)	207 (4.3)	4788 (9.1)	2617 (5.0)	6075 (20.1)	2718 (9.0)	9230 (14.6)	4276 (6.7)

Hemiplegia or paraplegia	25 (0.5)	3 (0.1)	101 (0.2)	38 (0.1)	252 (0.8)	230 (0.8)	421 (0.7)	325 (0.5)
Metastatic solid tumour[e]	0 (0.0)	0 (0.0)	0 (0.0)	0 (0.0)	0 (0.0)	0 (0.0)	0 (0.0)	0 (0.0)
Mild liver disease	99 (2.1)	52 (1.1)	1424 (2.7)	592 (1.1)	1454 (4.8)	769 (2.6)	4214 (6.6)	1179 (1.9)
Moderate or severe liver disease	5 (0.1)	1 (0.02)	69 (0.1)	22 (0.04)	89 (0.3)	74 (0.25)	227 (0.4)	96 (0.2)
Myocardial infarction	28 (0.6)	8 (0.2)	228 (0.4)	106 (0.2)	501 (1.7)	355 (1.2)	940 (1.5)	430 (0.7)
Peptic ulcer disease	13 (0.3)	5 (0.1)	232 (0.4)	76 (0.1)	175 (0.6)	91 (0.3)	529 (0.8)	160 (0.6)
Peripheral vascular disease	73 (1.5)	21 (0.4)	579 (1.1)	294 (0.6)	1249 (4.1)	614 (2.0)	1597 (2.5)	785 (1.2)
Renal disease	88 (1.8)	33 (0.7)	942 (1.8)	310 (0.6)	1128 (3.7)	572 (1.9)	1540 (2.4)	751 (1.2)
Rheumatic disease	48 (1.0)	33 (0.7)	876 (1.7)	447 (0.9)	325 (1.1)	246 (0.8)	1158 (1.8)	549 (0.9)
Non-CCI comorbidities, n (%)[d]								
Pain	1734 (36.3)	1215 (25.5)	26,064 (49.4)	16,038 (30.4)	14,486 (48.0)	9819 (32.5)	40,721 (64.2)	20,080 (31.6)
Substance use disorders[f]	540 (11.3)	39 (0.8)	5570 (10.6)	357 (0.7)	7290 (24.2)	1959 (6.5)	17,049 (26.9)	3199 (5.0)
Nicotine dependence	534 (11.2)	81 (1.7)	4406 (8.4)	949 (1.8)	9952 (33.0)	3820 (12.7)	22,134 (34.9)	6989 (11.0)

Anxiety disorders	1409 (29.5)	289 (6.1)	21,942 (41.6)	4044 (7.7)	8763 (29.0)	2896 (9.6)	29,037 (45.8)	6352 (10.0)
Depressive disorders	1146 (24.0)	224 (4.7)	14,641 (27.7)	3188 (6.0)	8223 (27.3)	2908 (9.6)	20,820 (32.8)	6297 (9.9)
Posttraumatic stress disorder	269 (5.6)	15 (0.3)	4638 (8.8)	245 (0.5)	2997 (9.9)	458 (1.5)	12,986 (20.5)	1040 (1.6)
Attention-deficit/hyperactivity disorder	274 (5.7)	100 (2.1)	7760 (14.7)	944 (1.8)	1338 (4.4)	391 (1.3)	8088 (12.8)	912 (1.4)
Personality disorders	163 (3.4)	6 (0.1)	2103 (4.0)	35 (0.1)	1871 (6.2)	71 (0.2)	4557 (7.2)	157 (0.3)
Medications, n (%)								
Antipsychotics	3735 (78.6)	20 (0.4)	25,596 (48.5)	315 (0.6)	24,684 (81.8)	772 (2.6)	36,126 (56.9)	1392 (2.2)
Antidepressants	2193 (46.0)	511 (10.7)	32,516 (61.6)	7423 (14.1)	15,536 (51.5)	4766 (15.8)	39,749 (62.6)	9776 (15.4)
Mood stabilizers	1476 (30.9)	135 (2.8)	34,479 (65.3)	1888 (3.6)	11,593 (38.4)	3117 (10.3)	35,062 (55.3)	5677 (9.0)
Anticonvulsants	1858 (38.9)	231 (4.8)	35,006 (66.3)	3245 (6.2)	13,447 (44.6)	3982 (13.2)	37,396 (58.9)	7268 (11.5)
Anxiolytics	1077 (22.6)	187 (3.9)	16,817 (31.9)	2840 (5.4)	8216 (27.2)	2333 (7.7)	25,459 (40.1)	4833 (7.6)
Sedatives and hypnotics	283 (5.9)	76 (1.6)	6025 (11.4)	1061 (2.0)	2322 (7.7)	579 (1.9)	6868 (10.8)	1134 (1.8)

[a]Race not available in the Commercial database.
[b]Regional division not available in the Medicaid database.
[c]CCI is a weighted score based on the number and the seriousness (scored 1–6) of comorbid diseases; higher scores are associated with a greater risk of mortality [1].

[d]Comorbidities were assessed by ≥1 ICD-9-CM or ICD-10-CM diagnosis code for the particular condition occurring during the baseline period, except for pain (≥2 diagnosis codes).
[e]Individuals with any cancer or metastatic cancer diagnoses during the analytic window were excluded from the analysis.
[f]Not including nicotine dependence.
CCI, Charlson Comorbidity Index.

REFERENCE

1. Charlson ME, Pompei P, Ales KL, MacKenzie CR. A new method of classifying prognostic comorbidity in longitudinal studies: development and validation. J Chronic Dis. 1987;40:373-83. 10.1016/0021-9681(87)90171-8.
