# Supplementary material for: Prescription opioid dispensing patterns among patients with schizophrenia or bipolar disorder
Source: BMC Psychiatry. 2024 Apr 2;24:244. doi: 10.1186/s12888-024-05676-5 (PMC10986122; doi:10.1186/s12888-024-05676-5)
Supplement: Supplementary file 6 — Additional File 6. Average Annual Days of Chronic Prescription Opioid Dispensing: (A) Commercial Database. (B) Medicaid Database. [file 12888_2024_5676_MOESM6_ESM.pdf]

Additional File 6. Average Annual Days of Chronic Prescription Opioid Dispensing: (A) Commercial database. (B) Medicaid database

A.

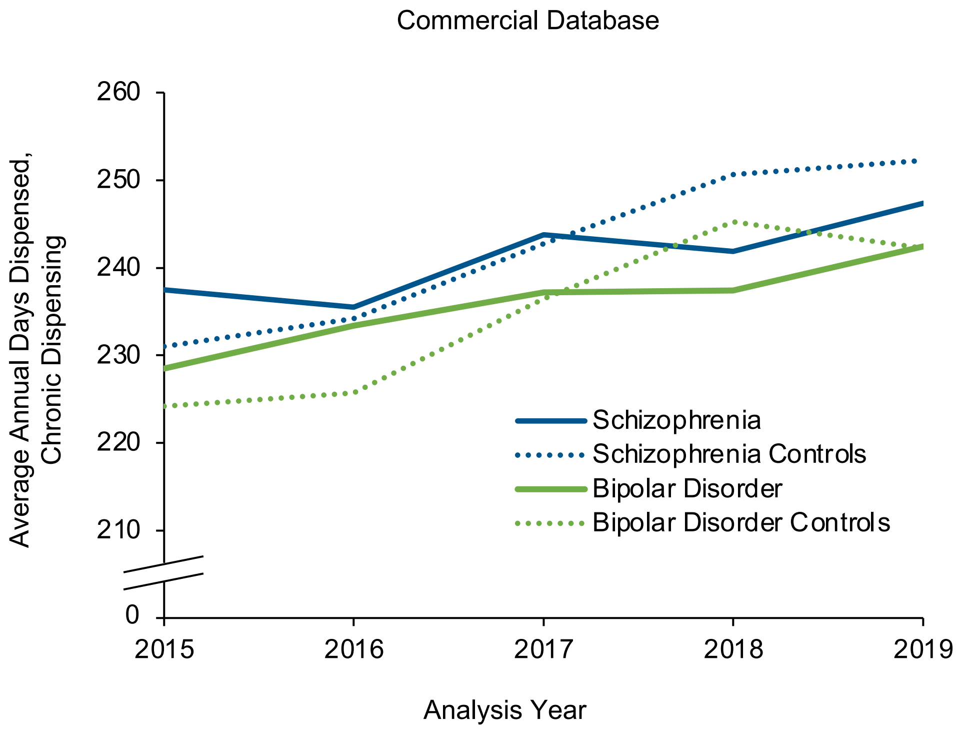

B.

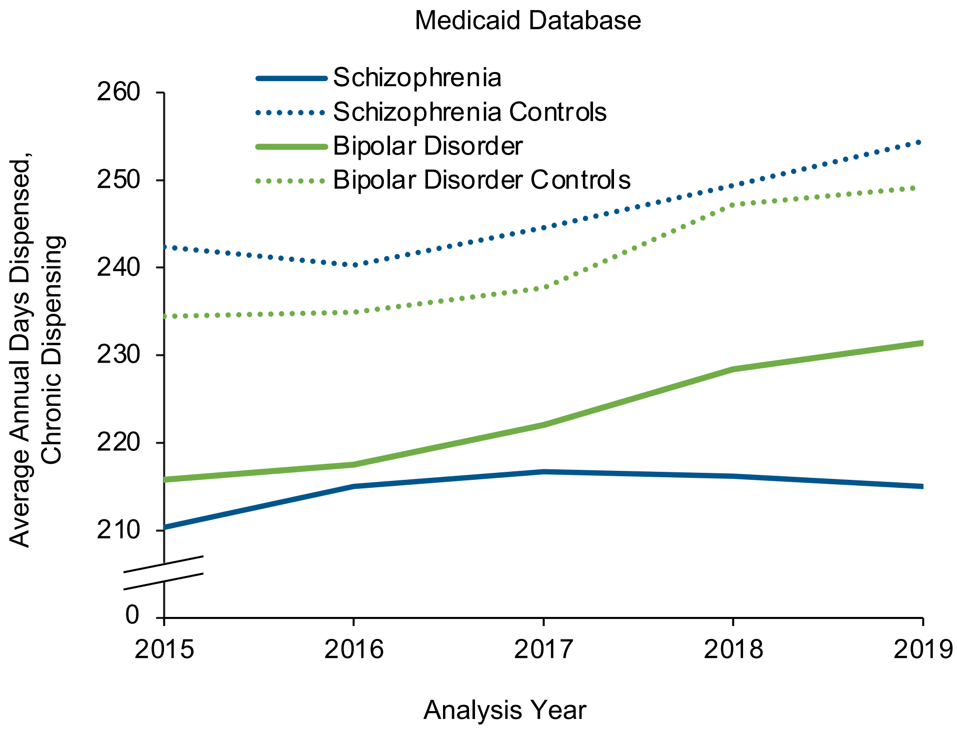

Medicaid Database
Schizophrenia
Schizophrenia Controls
Bipolar Disorder
Bipolar Disorder Controls
260
250
240
230
220
210
0
Average Annual Days Dispensed, Chronic Dispensing
2015
2016
2017
2018
2019
Analysis Year
